# Configurable Synaptic and Stochastic Neuronal Functions in ZnTe‐Based Memristor for an RBM Neural Network

**DOI:** 10.1002/advs.202405768

**Published:** 2024-09-05

**Authors:** Jungang Heo, Seongmin Kim, Sungjun Kim, Min‐Hwi Kim

**Affiliations:** ^1^ Division of Electronics and Electrical Engineering Dongguk University Seoul 04620 Republic of Korea; ^2^ School of Electrical and Electronics Engineering and Department of Intelligent Semiconductor Engineering Chung‐Ang University Seoul 06974 Republic of Korea

**Keywords:** neuromorphic system, OTS, RBM, stochastic neuron, synaptic devices

## Abstract

This study presents findings that demonstrate the possibility of simplifying neural networks by inducing multifunctionality through separate manipulation within a single material. Herein, two‐terminal memristor W/ZnTe/W devices implemented a multifunctional memristor comprising a selector, synapse, and a neuron using an ovonic threshold switching material. By setting the low‐current level (µA) in the forming process, a stable memory‐switching operation is achieved, and the capacity to implement a synapse is demonstrated based on paired‐pulse facilitation/depression, potentiation/depression, spike‐amplitude‐dependent plasticity, and spike‐number‐dependent plasticity outcomes. Based on synaptic behavior, the Modified National Institute of Standards and Technology database image classification accuracy is up to 90%. Conversely, by setting the high‐current level (mA) in the forming process, the stable bipolar threshold switching operation and good selector characteristics (300 ns switching speed, free‐drift, recovery properties) are demonstrated. In addition, a stochastic neuron is implemented using the stochastic switching response in the positive voltage region. Utilizing stochastic neurons, it is possible to create a generative restricted Boltzmann machine model.

## Graphical abstract

An Ovonic threshold switching material based multifunctional memristor device is presented. The two‐terminal multifunctional memristor is expected to function as a selector in memory arrays, a synaptic device and a stochastic neuron in neuromorphic systems by setting the appropriate operating current level in the forming process. Utilizing the stochastic neurons, a restricted Boltzmann machine model is created.

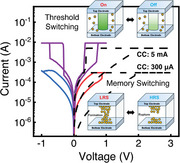

## 1 Introduction

There has been an increase in the demand for large‐scale data processing in a variety of applications following the fourth industrial revolution, including big data, artificial intelligence, and the Internet of Things. However, there are challenges associated with the processing of vast amounts of data in existing computer systems given their von Neumann structures. This is because the gap in the speed for data processing speed between the computer processing unit and memory causes a bottleneck.^[^ ^1^, ^2^, ^3^ ^]^ It causes an increase in power consumption and requires significant processing time. To solve this problem, a novel structured neuromorphic computing system was proposed in previous studies.^[^ ^4^, ^5^ ^]^ A neuromorphic computing system, which is based on artificial neural networks, is a promising replacement candidate architecture for the von Neumann structure. Neural networks attempt to emulate the human brain which is composed of an intricate network of connections between multiple neurons and synapses, by utilizing artificial synapses and neurons.^[^ ^6^, ^7^ ^]^ Neurons accumulate inputs from synapses and generate electrical signals when they reach or surpass a specific threshold.^[^ ^8^, ^9^ ^]^ While neurons play generate and transmit signals, synapses store or process incoming signals. In this case, data processing and memory can be performed by altering synaptic weights based on the intensity of incoming stimuli.^[^ ^10^, ^11^, ^12^ ^]^ As a result, applying a neural network composed of neurons and synapses allows for low‐power consumption and parallel data processing.

Several studies have used memristors to create artificial synapses or artificial neurons to implement in‐memory computing by exploiting the analog memristor resistance changes. For example, Kim et al. proposed an HfO_x_/AlO_x_‐based, vertically stacked, resistive random‐access memory (VRRAM),^[^ ^13^ ^]^ and implemented a synapse by utilizing the analog resistive changes in the metal oxide induced by the mobility of oxygen vacancies. Zhang et al. fabricated a memristor with a simple Pt/FeO_x_/Ag structure and successfully implemented LIF neurons by adjusting the electrical pulse interval and number of pulses.^[^ ^14^ ^]^ Tuma et al. leveraged the inherent randomness of phase‐change memory (PCM) by utilizing the phase‐changing material Ge_2_Sb_2_Te_5_.^[^ ^15^ ^]^ In this study, stochastic neurons were implemented using PCM characteristics, where state changes occur probabilistically through the adjustment of pulse intervals and the number of electrical pulses. Consequently, previously conducted research has used certain compounds to simulate synapses or neurons. However, while many studies have focused on memristor devices serving synapses or neurons, only a few studies have evaluated devices capable of fulfilling multiple functions. Wang, Tianyu, et al. manufactured the memristor device that using CNT fibers and the 2D materials MoS_2_.^[^ ^16^ ^]^ The device has volatile and nonvolatile characteristics which implemented by adjusting the operating current level of the device, allowing it to function as a synapse or LIF neuron. Also, John, Rohit Abraham, et al. reported that the memristor using perovskite materials has also multiple switching characteristics.^[^ ^17^ ^]^ Like the previous paper, this device exhibits the same two operating characteristics depending on the current compliance (CC). The switching mechanism is explained through the reaction between perovskite materials and Ag ion. In this device, these two characteristics were used to form an artificial neural network (ANN). In previous studies, multi‐layers were deposited using the ALD process for switching layers, along with materials such as 2D materials or perovskite. This approach has the disadvantage of requiring various equipment for device manufacturing. If a single material can perform multiple functions at once, it is simple to manufacture neural networks that require synapses and neurons. Therefore, the development of simple structure multifunctional memristive hardware is required. Existing studies have implemented selectors using ZnTe, an ovonic threshold switching (OTS) materials, and other studies have reported that resistive switching with ZnTe materials is possible.^[^ ^18^, ^19^, ^20^, ^21^ ^]^ Based on previous research, it is evident that ZnTe can facilitate both resistive switching and threshold switching. This suggests that ZnTe is a material that can be implemented to function as both a synapse and a neuron.

**Figure 1:**
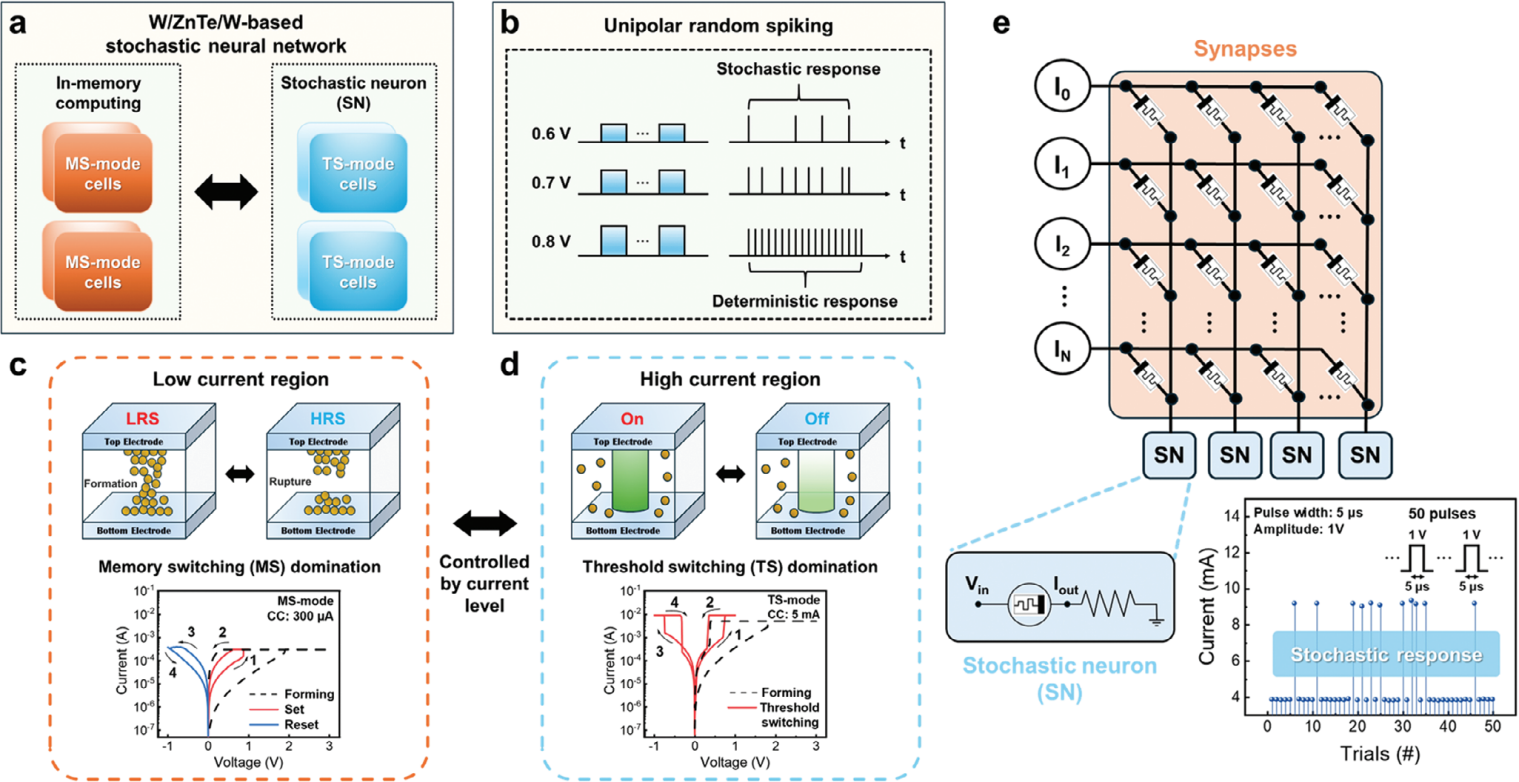
a) A stochastic neural network based on the same W/ZnTe/W devices. b) Description of the different responses based on the pulse amplitude. c) Memory switching operation in the low‐current synaptic regions (µA unit) for synapses, and d) threshold switching operation in the high‐current region (mA unit) for stochastic neurons. e) Integration of synapses and neurons in a fully memristive neural network.

In this study, we developed a device that can simultaneously implement the selector, synapses, and neurons using OTS materials, as depicted in **Figure** **1**a. It can serve as an effective selector by meeting the fundamental requirements of an increased switching speed (290 to 320 ns), short recovery and wait times (50 ns or less), and stable operations in both directions, as shown in Figure 1d. Furthermore, additional memory switching (MS) behavior was observed in the low−current region, as illustrated in Figure 1c, due to oxygen bonding with OTS materials generated during the manufacturing process. The physical origin of the MS operations was investigated using transmission electron microscopy (TEM), energy‐dispersive X‐ray (EDS) spectroscopy, and X‐ray photoelectron spectroscopy (XPS). We present an experimental demonstration of a hardware implementation of synapses using a nonvolatile ZnTe‐based memristor that successfully emulates synaptic plasticity. We utilize synaptic roles to achieve modified National Institute of Standards and Technology database (MNIST) classification accuracy, obtaining results up to 90.55%. It is also confirmed that the device can be used as a synapse based on its MS characteristics. Lastly, it was integrated as a neuron within a restricted Boltzmann machine (RBM) model, a generative model type, leveraging its threshold switching (TS) operation and stochastic response characteristics within a specific pulse voltage range, as shown in Figure 1b. When the RBM neural network was implemented using stochastic neurons, the ability to generate new MNIST images reflecting the characteristics of the original MNIST image was successfully confirmed. Furthermore, it was confirmed that the MNIST image classification accuracy obtained by employing the reconstruction of MNIST images ranged between 90% and 92%. The results indicate that a memristor or any other device with a stochastic response can effectively function as a stochastic neuron. Thus, a more competitive neural network can be achieved by utilizing a single OTS material to create a multifunctional device capable of performing the functions of a selector, synapse, and neuron, as depicted in Figure 1e.

## 2 Results and Discussion

### 2.1 Experimental Demonstration of the ZnTe Memristor

**Figure 2:**
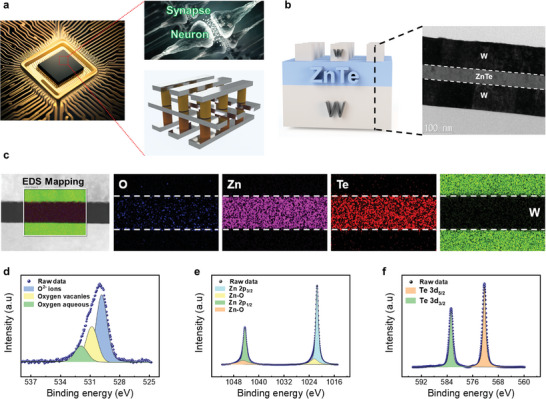
a) Concept of the multifunctional ZnTe‐based memristor. b) Schematic of the device and the cross‐sectional transmission electron micrograph image, and c) energy dispersive spectroscopy (EDS) maps of O, Zn, Te, and W. X‐ray photoelectron spectroscopy core‐level spectra of the d) O 1s peak in ZnTe layer, e) Zn 2p, and f) Te 3d.

The device, as illustrated in **Figure** **2**a, is capable of functioning as a selector, neuron, or synapse. Its ability to switch roles via external electrical stimuli offers a significant hardware advantage for neural network implementation. We investigated the physical and chemical origins of the multifunctional properties. First, the cross‐sectional TEM image (shown in Figure 2b) reveals the device's simple two‐terminal structure, which includes W top and bottom electrodes with thicknesses of 100 nm, and a ZnTe layer with an approximate thickness of 50 nm. Figure 2c presents the initial state EDS elemental mapping profiles of the device, confirming the presence of the elements O, Zn, Te, and W in each layer. The oxygen in the ZnTe layer is evenly distributed, as shown in Figure 2c. As a similar phenomenon was observed in previous publications, it was assumed that the induction of redox reactions during device fabrication is what led to the detection of a trace amount of oxygen.^[^ ^22^, ^23^ ^]^ To investigate the oxygen chemical bonds in ZnTe, XPS analysis was performed. The XPS plot of the O 1s peak spectra shown in Figure 2d, indicates the presence of oxygen ions (O^2−^), oxygen vacancies, and aqueous oxygen in the ZnTe layer. To confirm the oxidation of the ZnTe layer, O 1s core‐level high‐resolution XPS spectra were fitted in Figure 2d with three peaks denoted as oxygen ions, oxygen vacancies, and aqueous oxygen.^[^ ^24^, ^25^, ^26^ ^]^ The peak at 530.0 eV (related to Zn−O or Te−O) emerged owing to the presence of the ZnTe layer.^[^ ^27^ ^]^ Figure 2e displays two small intensity oxygen peaks combined with Zn at 1022.0 eV and 1045.0 eV.^[^ ^28^, ^29^ ^]^ Conversely, Figure 2f shows no oxygen peaks combined with Te, indicating that Zn was combined with oxygen in the ZnTe layer. When metal oxide was used as an insulator in RRAM, MS behavior was made possible by the oxygen vacancies, which are linked to the peak at 531.0 eV.^[^ ^30^, ^31^ ^]^ Based on the EDS and XPS data analysis, the ZnTe‐based memristor shows promising potential for MS behavior. Through the physical and chemical analysis, we included a description of the two switching mechanisms of our device, as illustrated in Figure S3 (Supporting Information).

### 2.2 Confirmation of Potential as a Selector

**Figure 3:**
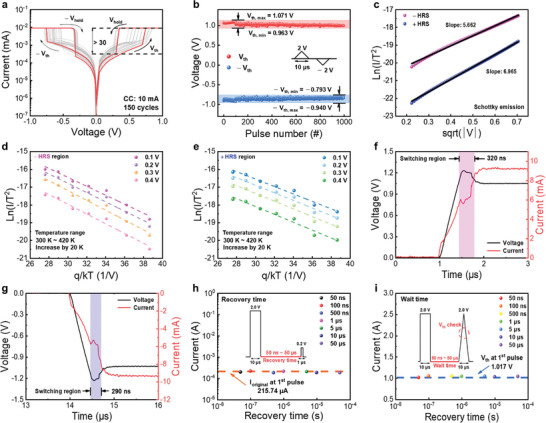
Electrical and operational characteristics of ZnTe‐based selector. a) Typical I–V curves (150 cycles) in bipolar threshold switching operational mode. b) Pulse endurance test. c) Fitted results of Schottky emission in positive and negative HRS. Analysis of Schottky thermal conduction at various temperatures in the d) negative and e) positive regions. f) Positive and g) negative switching times. Properties of the ZnTe‐based selector; current responses as functions of h) recovery time and i) drift‐free (wait time).

Previous research has shown that selectors made from OTS materials exhibit increased switching speeds, low‐energy consumption, and high‐endurance characteristics.^[^ ^32^, ^33^, ^34^ ^]^ Furthermore, memristor devices composed of OTS materials typically exhibit TS behavior and low V_on_ characteristics. We demonstrated that the ZnTe‐based selector possesses the characteristics. Typical I–V hysteretic curves (>150 cycles) of the device with a compliance current (CC) of 10 mA are shown in **Figure** **3**a. After the forming process, the device configured with 10 mA CC follows a bipolar TS behavior. I–V curves (spanning 20 cycles) were measured in 20 distinct cells to confirm the cell‐to‐cell variation for this device. The results are displayed in Figure S1 (Supporting Information). Although the three cells exhibit ohmic characteristics during the cycle, it is evident that the TS operation is performed with an on/off ratio of ≥10. Additionally, it is observed that while the value of V_hold_ remains relatively constant, the value of V_th_ varies randomly in each cycle. Given the random nature of the V_th_ in the I–V curve, the pulse endurance of the device was tested >150 times with the pulse amplitude set to 2 V. Figure 3b shows the voltage results of the device for 1000 pulses. The stability of the TS operation is indicated by the V_th_ variation ranging from a minimum of 0.963 V to a maximum of 1.071 V, and the ‐Vth variation ranging from −0.793 to −0.940 V.

To examine the conduction mechanism of the selector, the I−V curves at positive and negative high resistance states (HRSs) were fitted and interpreted according to the Schottky emission conduction mechanism in conjunction with fitted plots and Schottky slope values for each state, as shown in Figure 3c. The linear fittings of ln(I/T^2^) versus sqrt(V) suggest that carrier transport may predominate in the case of Schottky emission. The conduction formula is expressed based on Equation (1),^[^ ^35^ ^]^

(1) $$J\;=\;A^{*}T^{2}exp\left[\frac{-q\left(\varphi_{b}-\;\sqrt{qE/4\pi \varepsilon_{0}\varepsilon_{r}}\right)}{k_{b}T}\right]$$

where the effective Richardson constant is represented by A*, *T* is the absolute temperature in Kelvin, *q* is the elementary charge, φ_*b*_ is the Schottky barrier height, ε_0_ is free‐space permittivity, ε_0_ is relative permittivity, *k_b_* is the Boltzmann constant, and *d* is the Schottky barrier distance. Additionally, *A* is the effective conduction area, and *J* is the current density, which is expressed as I/A. The following equation replaces the Schottky emission conduction formula to evaluate the ln(I/T^2^) versus sqrt(V) fitting curve in Equation (2),^[^ ^36^ ^]^

(2) $$\ln\left(\frac{I}{T^{2}}\right)=\frac{q\sqrt{\frac{qE}{4\pi \varepsilon_{0}\varepsilon_{r}d}}}{k_{b}T}\;\sqrt{V}-\frac{q\varphi_{b}}{k_{b}T}+\ln AA^{*}$$

There is a linear relationship with this equation between sqrt(V) and ln(I/T^2^). Consequently, as shown in Figure 3c, we plotted the I−V curves for both states using the sqrt(V) on the x‐axis and ln(I/T^2^) on the y‐axis; each state exhibits a linear relationship. Figure 3d,e depict a corresponding relationship between the voltage and current density of the HRS, which is dependent on temperature. The temperature was adjusted for the device at 20 K intervals, and ranged from 300 to 420 K. It can be concluded that the current conduction is attributed to Schottky thermal conduction as the I–V fitting and the currents are proportionate to temperature.

One crucial feature of ZnTe‐based selector devices is their capability for fast switching. To evaluate the transient response of the ZnTe selector, the current was measured during a voltage pulse with an amplitude of 1.2 V, rising and falling times of 500 ns, and a 10 µs pulse width, as shown in Figure 3f,g. The switching time was calculated by identifying the point where the current changes instantaneously when a pulse is applied. Based on this definition, the switching time was obtained by comparing the time before and after the current rises rapidly upon applying a square pulse. The peak current in the ZnTe selector occurred 320 ns after the maximum voltage of 1.2 V was reached. Upon discontinuation of the applied voltage, the device switched to the off state within 290 ns. The switching energy can be defined as the value consumed from the off state to the on state and then back to the off state. The aforementioned can be formulated using Equation (3),^[^ ^37^ ^]^

(3) $$E_{sw}=\int_{t_{i}}^{t_{f}}V_{AK}\left(t\right)i_{s}\left(t\right)dt\;$$

where *t_i_* is the time immediately before the flow of current through the device, whereas *t_f_* represents the time after which no current flows through the device. Using the equation provided, the calculated switching energy is 548.10 nJ in the positive region (*t_i_* is 1 µs, *t_f_* is 10 µs) and 545.63 nJ in the negative region (*t_i_* is 1 µs, *t_f_* is 10 µs). As each state has a similar switching speed, the energy consumed is similar (≤550 nJ).

To measure the recovery time, we applied a large voltage (2.0 V) that can change the device from the off‐state to the on‐state and set the recovery time range from 50 µs to 50 ns. Raw measurement data are shown in Figure S4 (Supporting Information); currents were recorded at 0.2 V. A consistent current of ≈215 µA was detected within a recovery period of 50 ns. Figure 3h illustrates that the selector can transition from an ohmic state to an insulating phase in less than 50 ns. To assess drift‐free operations, we also applied the same large voltage pulse (2.0 V) across various wait times from 50 µs to 50 ns; raw data measurements are shown in Figure S5 (Supporting Information). Wait time is a measurement method to check whether the V_th_ of the device changes due to the previously applied pulse. Generally, a slope‐shaped pulse is used to obtain V_th_. Therefore, V_th_ is obtained by applying a triangular pulse. During the wait time interval, no voltage was applied. Subsequently, a triangular pulse was applied to evaluate the effects of the AC pulse on the V_th_ of the device. As shown in Figure 3i, even if a 50 ns wait time pulse is applied to the device, the V_th_ values fluctuate in the vicinity of 1.0 V, indicating that this device has drift‐free properties. The observed non‐measurable drift is critical because it provides an opportunity to improve the performance of cross‐point arrays or broaden the range of drift‐sensitive applications.

### 2.3 Artificial Synapses Based on RS‐Mode in Low‐Current Regions

An RRAM device that emulates synapses typically operates by modifying its resistance state based on the movement of metal ions or oxygen vacancies within the RRAM device.^[^ ^38^, ^39^, ^40^, ^41^ ^]^ Metal oxides such as HfO_x_, ZrO_x_, and ZnO_x_, are employed as insulators in RRAM devices in which the switching mechanism is determined by the movement of oxygen vacancies.^[^ ^42^, ^43^, ^44^ ^]^ The alteration in resistance state is regulated by external stimuli, such as voltage pulse or light illumination to emulate synaptic activity.^[^ ^6^, ^45^, ^46^, ^47^, ^48^ ^]^

**Figure 4:**
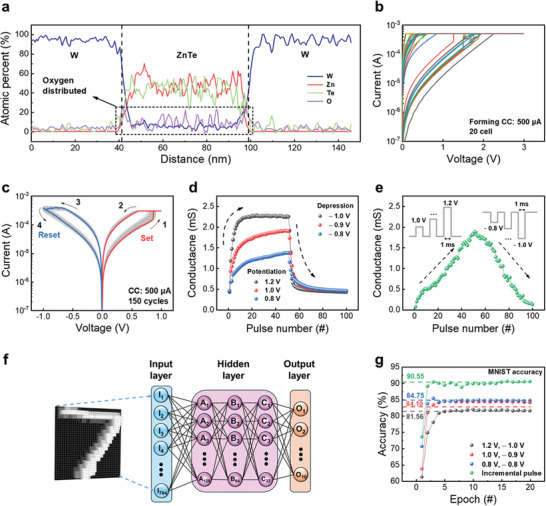
a) EDS elemental line profile of the W/ZnTe/W device. The oxygen is evenly distributed in the ZnTe layer. b) Forming curve in 20 random cells with a low‐current compliance current (CC). c) Typical I–V curves after the forming process (150 cycles). d) Multilevel potentiation and depression characteristics following the application of the same pulse. e) Linear conductance change using an incremental pulse. f) Framework of neural networks used for simulations of modified National Institute of Standards and Technology database (MNIST) pattern recognition. g) The accuracy of pattern recognition over twenty consecutive epochs at incremental and three other specific identical pulses.

Based on EDS mapping (Figure 2c), XPS data (Figure 2d), and the EDS line profiles (**Figure** **4**a), it was confirmed that oxygen was evenly distributed within the ZnTe layer and chemically reacted with Zn to form a Zn−O bond. This enabled the induction of MS characteristics in OTS materials. The top electrode was assigned to positive voltages during I–V measurements, and the bottom electrode was grounded. Figure 4b displays typical I–V curves for the forming process across 20 randomly selected W/ZnTe/W cells measured at low‐current levels in µA units. A direct current (DC) voltage sweep with a CC of 500 µA indicates a continuous current increase during the forming process. In the majority of the cells, the forming voltage ranged from 1 to 2 V; this means it consumes low levels of power as it does not require a high voltage during the forming process. After the forming process, consecutive I–V curves of memristor devices were obtained by applying positive and negative voltage sweeping sequences of V: 0→1.0, V: 0→−1.0 V. Figure 4c depicts the multiple switching cycles of the device. During the positive sweeping cycle, the device shows a gradual current increase up to 0.75 V followed by the triggering of a device transition from the HRS to the low‐resistance state (LRS), a phenomenon referred to as the “set” process. Even when a negative voltage is applied, the LRS is maintained until −0.75 V; immediately after this voltage, the device undergoes a transition from the LRS to the HRS, a procedure denoted as the “reset” process. The histograms of V_set_ and V_reset_ for the ZnTe‐based memristor device reveal broad distributions (ranging from 0.6 to 1.0 V) owing to the random formations of conductive filaments. These variations are also discovered across 20‐cell multiple switching cycles, as shown in Figure S2 (Supporting Information).

This device can be utilized to create a neuromorphic system, given the structural similarity of the conductive filament (connecting the top to the bottom electrodes) with that associated with the connection of pre‐ to post neurons, as illustrated in Figure S6 (Supporting Information). Synaptic plasticity, the process by which synaptic weight changes in response to external stimuli (input spikes), enables signal transfer between connected neurons. The input spikes at the pre neurons resemble the applied electric pulse at the top electrode, which trigger oxygen ion migration through the ZnTe layer, thereby causing a conductance change. In neuroscience, the form and timing of external stimuli affect synaptic plasticity.^[^ ^49^ ^]^ Hebbian theory extensively describes various types of plasticity, with representative synaptic behaviors, including potentiation and depression, paired−pulse facilitation/depression (PPF/PPD), potentiation/depression, spike−timing−dependent plasticity, spike−amplitude−dependent plasticity (SADP), and spike−number−dependent plasticity (SNDP).^[^ ^50^, ^51^ ^]^ Potentiation and depression are dynamic states that are manifested as changes in synaptic potential in response to synaptic spikes. Potentiation strengthens synaptic connectivity, enhancing signal transmission, whereas depression weakens it, reducing signal transmission. These mechanisms are applied in neuromorphic computing to improve the learning and reasoning abilities of artificial neural networks. Long‐term potentiation (LTP) and long‐term depression (LTD) are synaptic behaviors emulated by the multilevel characteristics of the W/ZnTe/W devices, as illustrated in Figure 4d. Potentiation is achieved using 50 identical pulses with voltage amplitudes ranging from 0.8 to 1.2 V in 0.2 V steps, and a constant pulse width of 1 ms. By contrast, depression is induced by employing 50 pulses (with a 1 ms pulse width) by reducing the pulse voltage from −1.0 to −0.8 V in 0.1 V steps. A large‐conductance change is induced by the first pulse when using a high‐voltage amplitude, which adversely affects the linearity of conductance updates.^[^ ^52^, ^53^ ^]^ Rather than applying a pulse of constant size, pulses of progressively increasing amplitude were used to reduce rapid conductance changes. Figure 4e illustrates that the use of an incremental voltage pulse scheme leads to a more linear conductance change. This method shows that conductance increases when the pulse voltage increases from 1.0 to 1.2 V in 4 mV increments following the application of 50 pulses. Conversely, conductance decreases when the pulse input voltage decreases from −0.8 to −1.0 V in 4 mV decrements following the application of 50 pulses. Figure S7 (Supporting Information) displays the results of 10 cycles that were conducted to verify the reproducibility of LTP/D obtained using incremental and identical pulses. These results show that the incremental pulse amplitude method is more efficient than the identical pulse amplitude method at modulating conductance values to achieve linear updates. The conductance values derived from both methods were analyzed using the MNIST dataset to determine their pattern recognition potential. Figure 4f shows the neural network architecture designed to evaluate the pattern recognition accuracy of MNIST data, where the synaptic weight incorporates normalized conductance from LTP/LTD, as shown in Figure S8 (Supporting Information). Each 28 × 28‐pixel region of an MNIST image was processed as an individual neuron in the input layer, yielding 784 neurons in total. The network features three hidden layers (with 128, 64, and 32 neurons) engineered to support the learning of complex patterns and characteristics.^[^ ^54^, ^55^ ^]^ Both the input and hidden layers employ the rectified linear unit function for activation, while the SoftMax function in the output layer computes the probability of the input image's classification, with accuracy ascertained by comparing the input image's label to the class with the highest probability. Figure 4g showcases the MNIST classification accuracy using synaptic weights derived from LTP/LTD conductance via the identical and incremental pulse methods. For the identical pulse method, the lowest accuracy of 81.56%, was noted with the highest voltage pulse (potentiation voltage: 1.2 V, depression voltage: −1.0 V), whereas the accuracies for the other two pulses were comparable at 84.10% and 84.75%. This suggests that gradual conductance changes offer more benefits than abrupt modifications. Additionally, the incremental pulse accuracy of 90.55% emphasizes that more linear modifications lead to higher accuracy compared with the identical pulse method.

Additionally, it is essential to store the synaptic weight for a certain period to use it as a synapse. If the device inputs the synaptic weight, it should maintain as long as possible. By measuring retention over 10 000 s, as shown in Figure S11 (Supporting Information), it can successfully perform the role of remembering the synaptic weight for an extended period.

**Figure 5:**
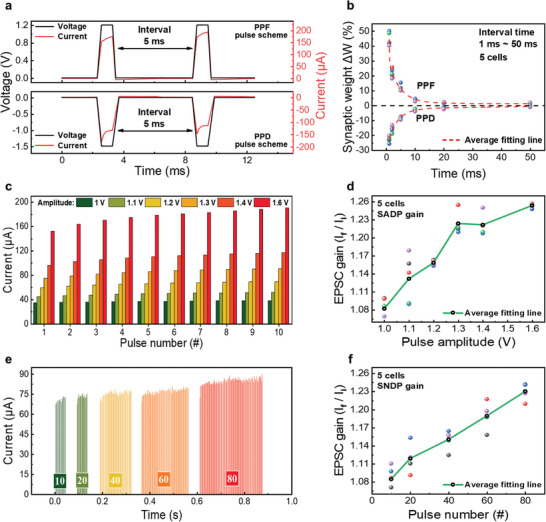
a) Paired‐pulse facilitation (PPF) and paired‐pulse facilitation/depression (PPD) schemes at 5 ms interval times. b) Synaptic weight based on the results of PPF and PPD measurements. Short‐term synaptic plasticity of the device with respect to c) spike‐amplitude‐dependent plasticity (SADP), and d) excitatory postsynaptic current (EPSC) gain based on SADP. e) Current difference was detected after the application of various pulse numbers ranging from 10 to 80, and f) EPSC gain based on spike‐number‐dependent plasticity.

PPF and PPD measurements were performed to emulate short‐term plasticity. **Figure** **5**a displays the transient current responses of the device when programming pulses are applied. The pulse conditions consist of voltage amplitudes of 1.2 V for set and −1.5 V for reset processes, with the same pulse width of 1 ms. Statistical analysis was used to assess the rate change trend between the first and second pulses in the two sequential inputs.^[^ ^56^ ^]^ Herein, the currents at the last point of the first pulse (I_1_) and last pulse (I_2_) were measured to determine the PPF or PPD value. As the interval time increases, the rate of current decay also increases, which resembles the short‐term memory effect presented in Figure 5b. The degree of change in synaptic weight can be expressed as,

(4) $$\Delta W=\;\frac{I_{2}-I_{1}}{I_{1}}\times 100\%$$

Thus, when the interval between the prepulse and postpulse increases, the postsynaptic potential decreases; this shows that the device may raise the synaptic weights in biological synapses by adjusting the interval between the two pulse stimuli. Furthermore, to ensure that the short‐term characteristics emerge more precisely, we also applied the PPF and PPD pulse schemes twice within all‐time intervals (1 ms to 50 ms), as illustrated in Figures S9 and S10 (Supporting Information). The measured results confirmed that the state of the device returned to its initial state which means the device was unaffected by the influence of previous pulses. As a result, the device could emulate the phenomena in which the human brain learns specific information so that as the time between learnings decreases, the stimulus applied to the brain becomes stronger.

Synaptic plasticity is viewed as a learning and memory function in neuromorphic systems achieved by varying synaptic weights. The conductance modulation characteristics of the device at various pulse amplitudes are shown in Figure 5c. Five different pulse trains are applied, each containing six pulse heights (1.0, 1.1, 1.2, 1.3, 1.4, and 1.6 V) with the same pulse width (100 µs), revealing the spike amplitude−dependent weight modulation characteristics. Figure 5e illustrates the SNDP characteristics of the W/ZnTe/W synaptic memristor. We applied five pulse numbers (10, 20, 40, 60, and 80 trials) with the same pulse width (10 µs). The behavior of the device appears to be dependent on the pulse number of conductance modulation, just as the SADP behavior. In artificial neural networks, signal transmission is indicated by the excitatory postsynaptic current (EPSC).^[^ ^57^ ^]^ Figure 5d,f respectively represent the EPSC gain versus the amplitudes of stimulus pulses and the number of stimulus pulses. Both EPSC gains can be calculated by using the following equation,

(5) $$EPSC\;gain=\frac{I_{f}}{I_{i}}$$

where *I_f_*, and *I_i_* represent the respective EPSC amplitudes after the final and initial pulses (see Figure 5d,f). The EPSC gain shows an increasing trend. This technique predicts the strength of synaptic memory depending on the number of repeats, similar to biological memory. These results show that mimicking the biosynaptic activity induced by the relaxation process is more clearly understood when the EPSC is increased by using more pulses at various pulse amplitudes.

### 2.4 Artificial Stochastic Neuron Based on TS‐Mode in High‐Current Regions

**Figure 6:**
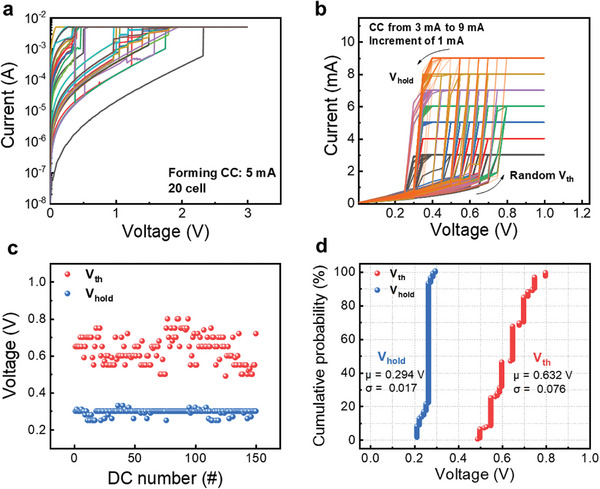
a) Forming curves in 20 random cells with a high CC (mA unit). b) Typical unipolar I–V curves in positive region with a CC ranging from 3 to 9 mA. c) I–V curves (150 cycles) showing the V_th_ and V_hold_ distributions. d) Cumulative probability results of V_th_ and V_hold_.

Two‐terminal memristors, including RRAM, phase‐change random access memory, and ferroelectric random access memory, exhibit probabilistic switching behavior.^[^ ^15^, ^58^, ^59^, ^60^ ^]^ Previous research often viewed this switching as a source of device variation, aiming to minimize it. However, when designing a stochastic neuron that operates probabilistically, the inherent probabilistic switching of memristors can be leveraged as a beneficial feature. In this study, stochastic firing characteristics within the positive voltage domain were exploited to simulate a stochastic neuron. This was achieved by applying a high CC during the forming process of the ZnTe‐based memristor, which induced threshold switching in the milliampere (mA) current range. Before inducing the TS operation, a forming process is necessary. **Figure** **6**a illustrates the standard I−V curves generated during the forming process for 20 randomly chosen W/ZnTe/W cells, with current measurements in mA. A DC positive voltage sweep and the application of a 5 mA CC demonstrated a consistent increase in the current throughout the forming process. The forming voltage for most cells ranged between 1.0 and 1.5 V. After the forming process, consecutive I−V curves of memristor devices were obtained by applying the positive voltage sweeping sequence of V: 0→1.0→0. The randomness of the V_th_ during the TS operation was confirmed by the I−V curve. Figure 6c,d show that the V_th_ occurs randomly within the range of 0.5 V to 0.8 V during DC operation. The variation (σ) in V_th_ is 0.076, which is greater than that observed in the V_hold_ (0.017), thus indicating a more probabilistic operation in the V_th_ range. As the voltage range operating probabilistically within this ZnTe‐based memristor has been identified, this device can be used as a stochastic neuron.

**Figure 7:**
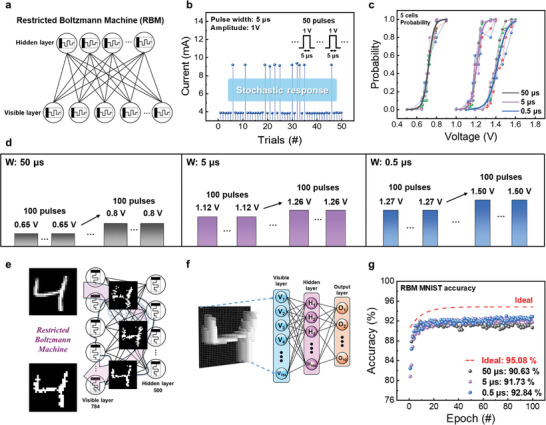
Implemented stochastic neurons using random threshold voltage distribution in a ZnTe‐based memristor. a) Schematic of restricted Boltzmann machine (RBM). b) The measured stochastic response obtained following the application of a fixed input positive pulse. Stochastic neuron switching for three distinct pulse widths (50 µs, 5 µs, and 0.5 µs). c) Probabilities of switching and d) pulse schemes. An MNIST handwritten digit “4” reconstructed by the generative RBM model. e) Image change according to training epoch. f) RBM model‐based neural network framework for simulating MNIST pattern recognition. g) Results of MNIST classification accuracies for three fixed pulse widths and general logistic function (ideal).

To utilize the stochastic neuron, it needs to be integrated into a neural network. A RBM serves as a prominent example of a network that incorporates stochastic neurons.^[^ ^61^, ^62^, ^63^ ^]^ In the RBM, “restriction” means there are no interconnections within a layer, allowing each node to act as a computation hub for making stochastic decisions about transmitting its input. A notable feature of RBM is its stochastic transfer functions, which allow the avoidance of local minima in optimization challenges. These machines are capable of learning from unlabeled data, forming a compact internal representation, and generating novel content through mimicry. The RBM is a stochastic binary neural network comprising two layers of stochastic neural units: a visible layer that receives input data, and hidden layers that connect to the visible units, as illustrated in **Figure** **7**a. Figure 7b demonstrates an experimental approach to verify the probabilistic operation of the device, based on a prior study.^[^ ^63^, ^64^, ^65^ ^]^ Figure 7b shows the stochastic fired response of ZnTe‐based stochastic neurons by applying 50 pulses with a duration of 5 µs and an amplitude of 1 V. A mixture of on‐ and off‐states is observed, indicating the device's stochastic response.

Using stochastic neurons in RBMs means that data transmission from the input to the next layer of neurons can be determined probabilistically. Therefore, for hardware implementation, the operational characteristics must vary probabilistically with external inputs. To verify this, pulse widths were categorized into three types: 50 µs, 5 µs, and 0.5 µs, as depicted in Figure 7d. For each pulse width, 100 pulses (with the same amplitude) were applied consecutively to determine if sections responded probabilistically or decisively based on the pulse amplitude. Following the application of a 50 µs pulse width, the on‐state was not observed for voltages <0.65 V, but it was predominantly induced for voltages >0.8 V. With a 5 µs pulse width, the device exhibited a probabilistic response for approximate voltage values between 1.1 and 1.25 V. For a pulse width of 0.5 µs, a probabilistic response was observed between ≈1.25 V and 1.5 V. Figures S12−S14 (Supporting Information) present the results for each pulse width experiment. The sigmoidal function, derived from the experimental results, is depicted in Figure 7c. This finding indicates that neurons with a sigmoidal‐type activation function can be constructed using a probabilistically behaving memristor. It was observed that an increase in pulse width led to device activation even with relatively lower voltage pulses. Additionally, the three distinct sigmoidal functions served as activation functions to determine the data transfer from the visible to the hidden layer (or vice versa) within the RBM.

Figure 7e illustrates a method to evaluate the reconstruction performance using the RBM generation model. This involves the processing of actual MNIST data through the RBM model and the comparison of the generated images with the original ones. According to Figure S15 (Supporting Information), the initially generated image differs from the original, but similarity increases significantly with more epochs. Furthermore, as shown in Figure 7f, the MNIST dataset was utilized by feeding images generated by the RBM model into a classification model. In the RBM model, which consists of a visible and a hidden layer, accuracy was determined by specifically applying the SoftMax function to the hidden layer. Different outcomes on the MNIST dataset were achieved by implementing the three distinct sigmoidal functions in the neurons of each layer.

As depicted in Figure 7h, the experiment utilizing the shortest pulse width achieved the highest accuracy of 92.84%; in contrast, the experiment with the longest pulse width recorded the lowest accuracy of 90.63%. If we use the general logistic sigmoid function (noted as ideal) for RBM MNIST pattern regeneration, we can achieve about 95% accuracy. The reduced accuracy can be attributed to the modulation of the sigmoid function.^[^ ^66^ ^]^ Basically, the RBM model is based on established theory and executed software simulations using the ideal sigmoid function. Therefore, using a shifted sigmoid for training the RBM model can lead to performance degradation. On the other hand, it is almost impossible to implement an ideal sigmoid as hardware and use it as an activation function. The ideal sigmoid function should have a 50% switching probability when the voltage is zero. This is not common because most memristor devices change their resistive state as voltage is applied, resulting in a shifted sigmoid function. Nevertheless, in the case of our device, we could not derive an ideal sigmoid function, but we subdivided the pulse width to derive as many sigmoid functions as possible. As a result, we aimed to approximate the performance of the ideal sigmoid function with only ≈2% difference in performance. Figure S16 (Supporting Information) presents the outcomes of five trials performed to validate the reproducibility of MNIST accuracy at various pulse widths. This variation indicates that the experimental approach influenced the RBM's performance. The consistent and stable outcomes suggest that the ZnTe‐based memristor RBM model is effective.

## 3 Conclusions

In this study, we fabricated a simple structure two‐terminal ZnTe‐based memristor and implemented a multifunctional memristor (selector, synapse, neuron) by controlling the current level in the forming process and DC voltage sweep. In high‐current regions (mA units), the DC I–V response, switching speed (≤300 ns), switching energy (≤550 nJ), recovery time (≤50 ns), and wait time (≤50 ns) were measured to ensure that the selector role was performed well. Conversely, in low‐current regions (µA units), the DC I–V responses, potentiation and depression, PPF, PPD, SADP, and SNDP were measured to ensure that the synaptic behavior was appropriate. Based on the synaptic plasticity,

MNIST pattern recognition accuracies up to 90.55% were obtained. In the positive high‐current region (mA unit), stochastic neurons were implemented by reflecting the stochastic switching characteristics of the memristor. Based on the stochastic neurons, a generative model RBM was created, and it was confirmed that the RBM MNIST pattern recognition accuracy increased from 90% to 92%. Therefore, by using one OTS material memristor to configure a neural network, the utilization of the hardware can be increased.

## 4 Experimental Section

The multifunctional memristor W/ZnTe/W devices were fabricated as follows. First, a 100‐nm‐thick W layer was deposited on a SiO_2_/Si substrate wafer via DC sputtering in the presence of gaseous N_2_ and Ar. ZnTe switching layers were subsequently deposited to an approximate thickness of 50 nm using sputtering at room temperature. After the switching layer deposition, the top electrode was patterned on the ZnTe layer by photolithography. A 100‐nm‐thick W top electrode was then deposited using DC magnetron sputtering followed by lift‐off. The comprehensive compositional and structural characterizations of the devices were performed by high‐resolution TEM, EDS, and XPS.

### Electrical Measurements

The I–V and transient curves of the ZnTe‐based memristor cells were obtained by measurements using a semiconductor parameter analyzer (Keithley 4200‐SCS and 4225‐PMU ultrafast current‐voltage module, Keithley Instruments, Cleveland, OH, USA). A bias was applied to the top W electrode, and the bottom W electrode was grounded.

### RBM Simulations

Two operations on a neural network were made possible by the RBM MNIST simulations. One of these used a SoftMax function as a classification model and demonstrated its ability to reproduce the original data (MNIST images) and the reproduced images. This allowed for the determination of its suitability for the RBM model. The RBM code was created by considering the previously established RBM theory.

RBM was made up of two layers. As shown in Figure 7a, the visible layer (input layer) is the layer to which the training example is presented, and the hidden layer is the layer connected to the visible layer by symmetric synaptic weights. In other words, the features of the original data may be extracted through the visible and hidden layers. RBM is an energy‐based generative model, and the energy of visible and hidden layers is described as,^[^ ^67^, ^68^, ^69^, ^70^, ^71^, ^72^ ^]^

(6) $$E\left(v,\;h\right)=-\sum_{i\;\in \;visivle}a_{i}v_{i}\;-\sum_{j\;\in \;hidden}b_{j}h_{j}-\sum_{i,j}v_{i}h_{j}w_{ij}\;\;$$

where *v_i_* and *h_j_* are the binary states (zero or one) of visible units and hidden units, *a_i_* and *b_i_* are the corresponding biases and *w_ij_* is the synaptic weight between the visible and hidden units.

The energy function assigns probability to each pair of visible and hidden vectors in a modeled network as follows,

(7) $$p\left(v,\;h\right)\;=\;\frac{1}{Z}\;e^{-E\left(v,h\right)}\;\;\;\;\;$$

(8) $$Z=\sum_{v,\;h}e^{-E\left(v,h\right)}\;\;\;\;$$

where Z is a normalization factor.

To reduce energy in the network for a training example, adjust the weights and biases based on the probability assigned by the network. This energy optimization could be achieved by maximizing the log probability with respect to the weights as follows,

(9) $$\frac{\delta \;log\;p\left(v,h\right)}{\delta w_{ij}}\;=\;{〈v_{i}h_{j}〉}_{\mathrm{original}\;\mathrm{data}\;}-\;{〈v_{i}h_{j}〉}_{\mathrm{reconstruction}\;\mathrm{data}}$$

This forms a simple learning rule in log probability for training data. Weights can be updated by the following equation,

(10) $$\Delta \;w_{ij}=\;\varepsilon \left({〈v_{i}h_{j}〉}_{\mathrm{original}\;\mathrm{data}\;}-{〈v_{i}h_{j}〉}_{\mathrm{reconstruction}\;\mathrm{data}}\right)$$

where ε is the learning rate.

The probability that the binary state of the hidden unit *h_j_* has a value of “1” can be expressed as,

(11) $$p\left(\;h_{j}=\;1|\;v\right)=\;\sigma \;\left(b_{j}+\;\sum_{i}v_{i}w_{ij}\right)\;\;\;$$

where σ(.) is the logistic sigmoid function defined as

(12) $$\sigma \;\left(t\right)=\;\frac{1}{1+e^{-t}}$$

In this study, we used a shifted sigmoidal function that reflected the stochastic switching characteristics of the device instead of the general logistic sigmoid function, as shown in Figure 7c. The shifted sigmoid function (σ*(*t*)) can be expressed as follows,

(13) $$\sigma^{*}\;\left(t\right)=\;f_{N}\;=\frac{1}{1+e^{-d\left(V-V_{0}\right)}}$$

where f_N_ means the probability of the switching, *d* is the switching variability which reflects the slope of the shifted sigmoid function, and V_0_ is the voltage at which f_N_ is equal to 0.5.

Therefore, Equation (6) can be expressed as follows,

(14) $$P\left(\;h_{j}=\;1|\;v\right)=\;\sigma^{*}\left(b_{j}+\;\sum_{i}v_{i}w_{ij}\right)\;\;\;\;\;$$

For this reconstruction data, a single step of Gibbs sampling can be performed, where a single step of Gibbs sampling determines the states of hidden units using (9) and calculates the visible layer state using (10).

(15) $$P\left(\;v_{j}=\;1|\;h\right)=\;\sigma^{*}\left(a_{i}+\;\sum_{j}h_{j}w_{ij}\right)\;\;\;$$

The results illustrated in Figure S15 (Supporting Information) could be attained by the RBM neural network which uses Python code that incorporates the measured data and the equations.

## Conflict of Interest

The authors declare no conflict of interest.

## Supporting information

Supporting Information

## Data Availability

The data that support the findings of this study are available in the supplementary material of this article.
