# Supplementary material for: Configurable Synaptic and Stochastic Neuronal Functions in ZnTe‐Based Memristor for an RBM Neural Network
Source: Adv Sci (Weinh). 2024 Sep 5;11(42):2405768. doi: 10.1002/advs.202405768 (PMC11558158; doi:10.1002/advs.202405768)
Supplement: Supplementary file 1 — Supporting Information [file ADVS-11-2405768-s001.docx]

Supporting Information

**Configurable Synaptic and Stochastic Neuronal Functions in ZnTe-Based Memristor for RBM Neural Network**

Jungang Heo^†^, Seongmin Kim^†^, Sungjun Kim* and Min-Hwi Kim*


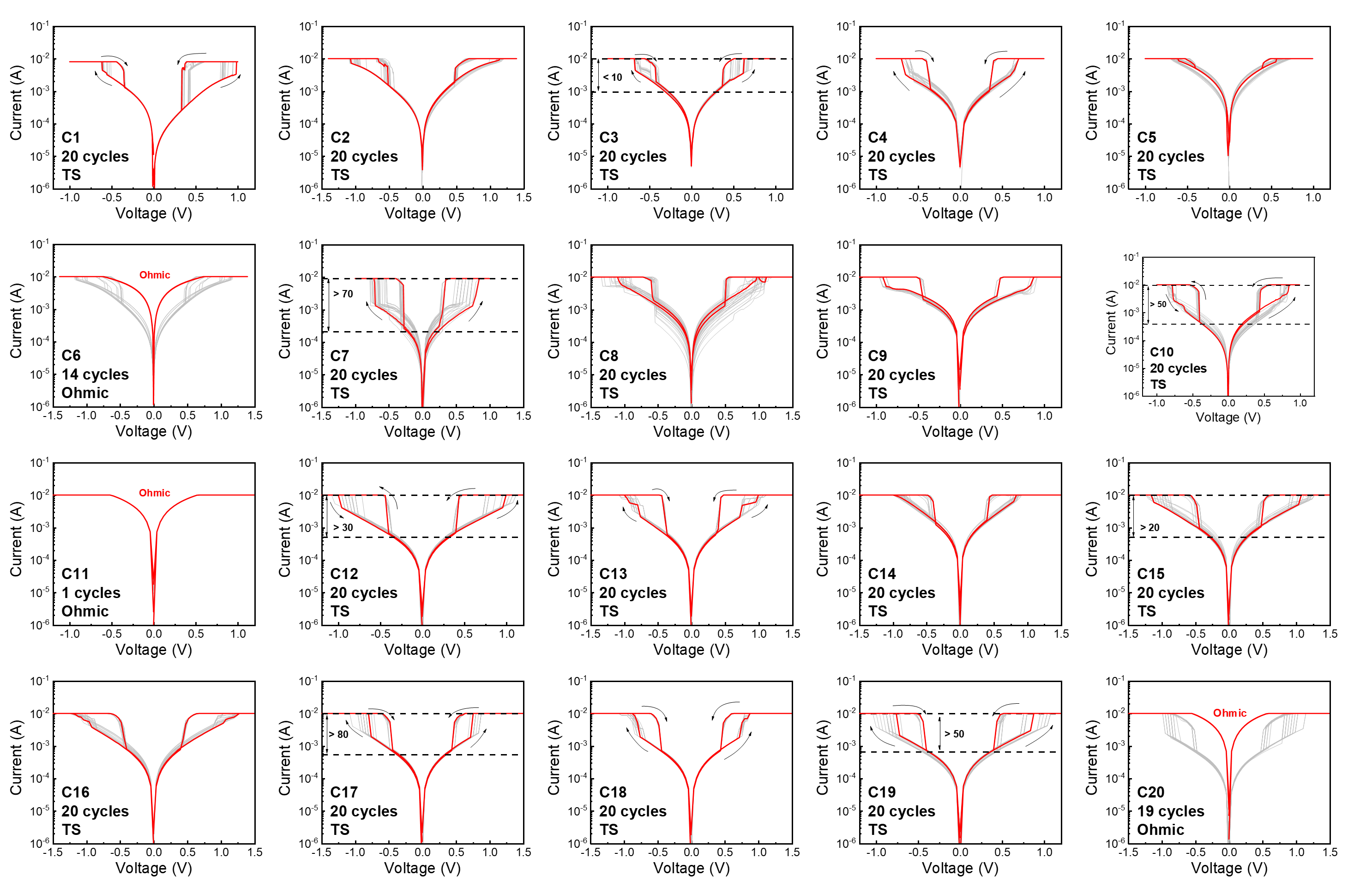


**Figure S1**. I-V curves 20 cycles in 20 random cells (operated at mA-level cc).


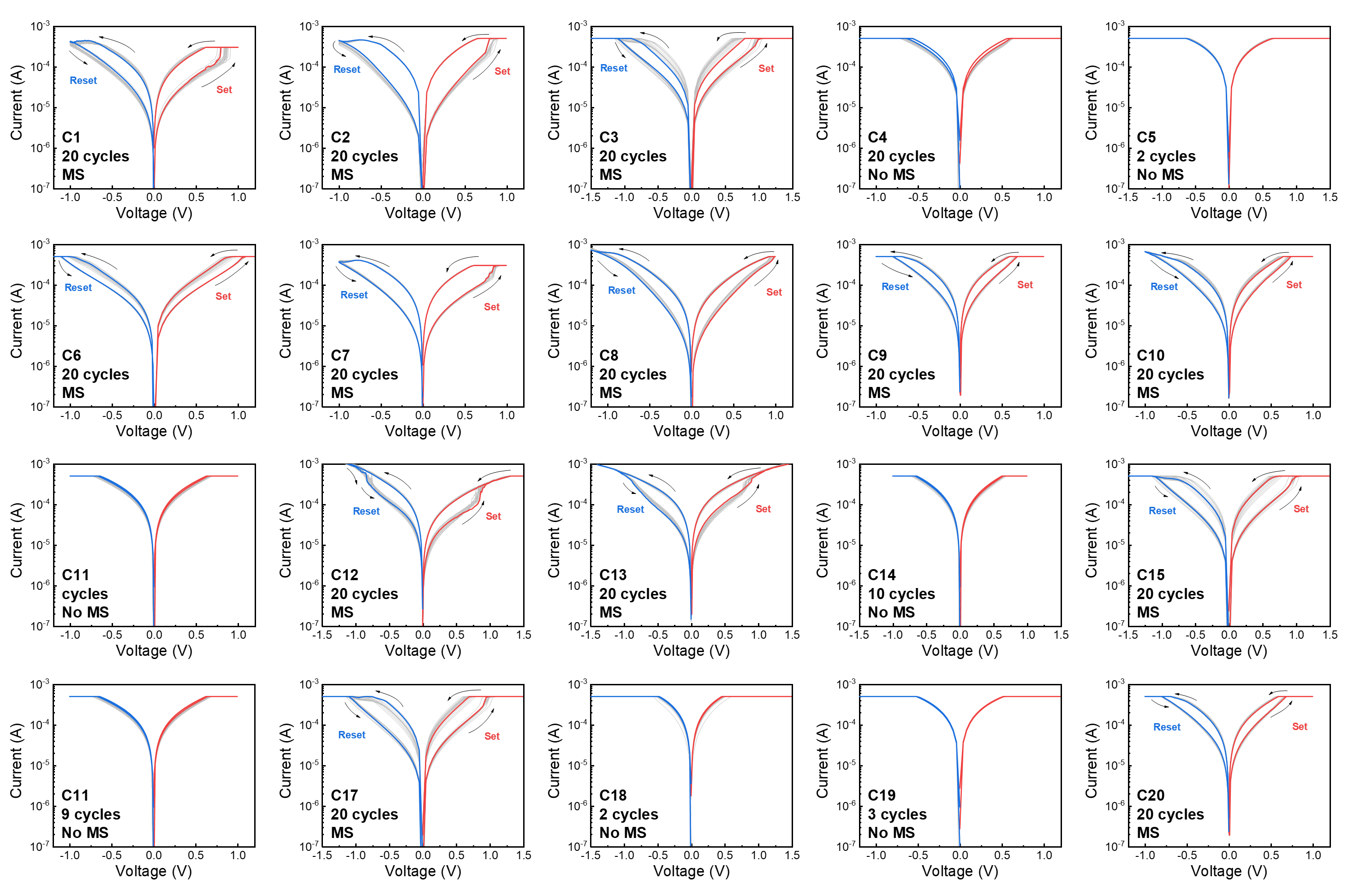


**Figure S2**. I-V curves 20 cycles in 20 random cells (operated at μA-level cc).


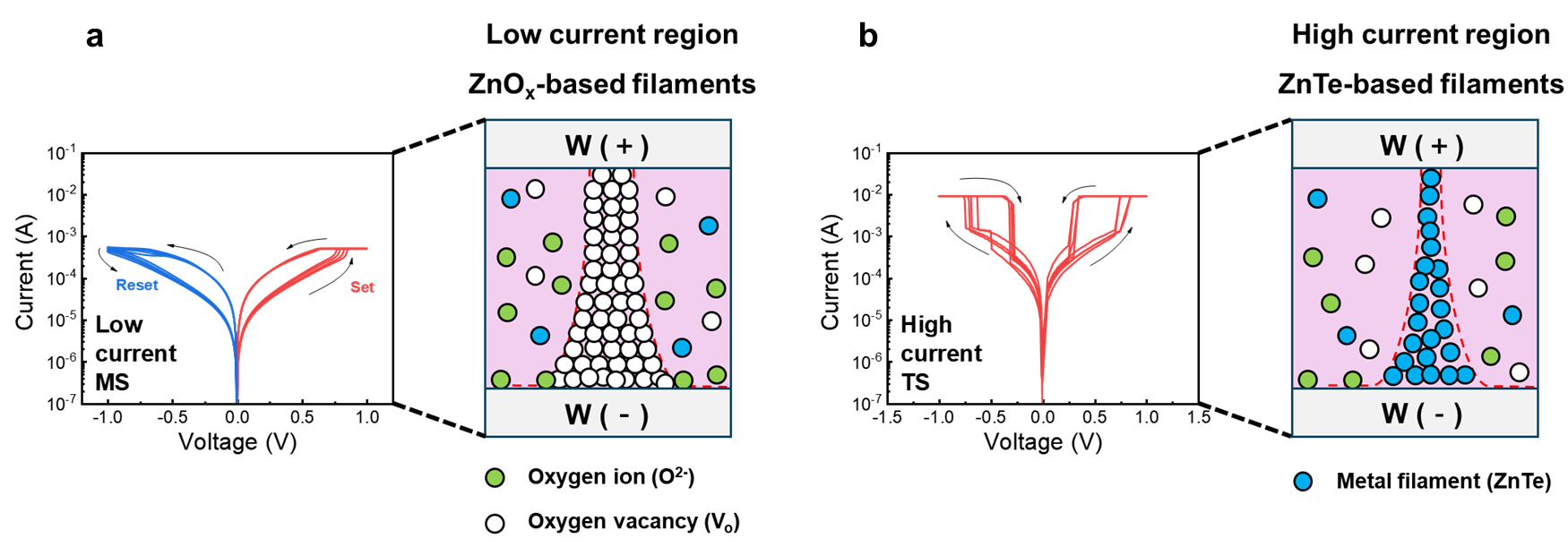


**Figure S3**. Switching mechanism within different current region a) Oxygen vacancy-based filaments, and b) Metal-based filaments.

When the forming process customizes the compliance current (CC), threshold switching (TS) and memory switching (MS) coexist in the ZnTe-based memristor. It is expected that the forming process determines the elements that form the conductive path of the memristor. In this memristor, oxygen vacancies form the conductive path if the forming process is conducted at a low current level (μA unit). Because of the uniformly distributed oxygen, ZnO_x_ is formed in the ZnTe layer, and oxygen vacancies are created within this ZnO_x_. Thus, when applied the positive voltage into the memristor, oxygen vacancies form conductive path that means low-resistance state (LRS) in device. On the contrary, applied the negative voltage into the device, oxygen vacancies combine with oxygen ions that rupture the conductive path, and make device in high-resistance state (HRS). In contrast, when the forming process is performed at a high current level (mA), it is assumed that the ZnTe (metal) forms the conductive path, resulting in threshold switching, which is an ovonic threshold switching (OTS) characteristic. Consequently, as shown in **Figure S**3, since both ZnTe and ZnO_x_ are present within the 50 nm thickness ZnTe layer, different filaments are formed by the CC, leading to either memory switching or threshold switching.


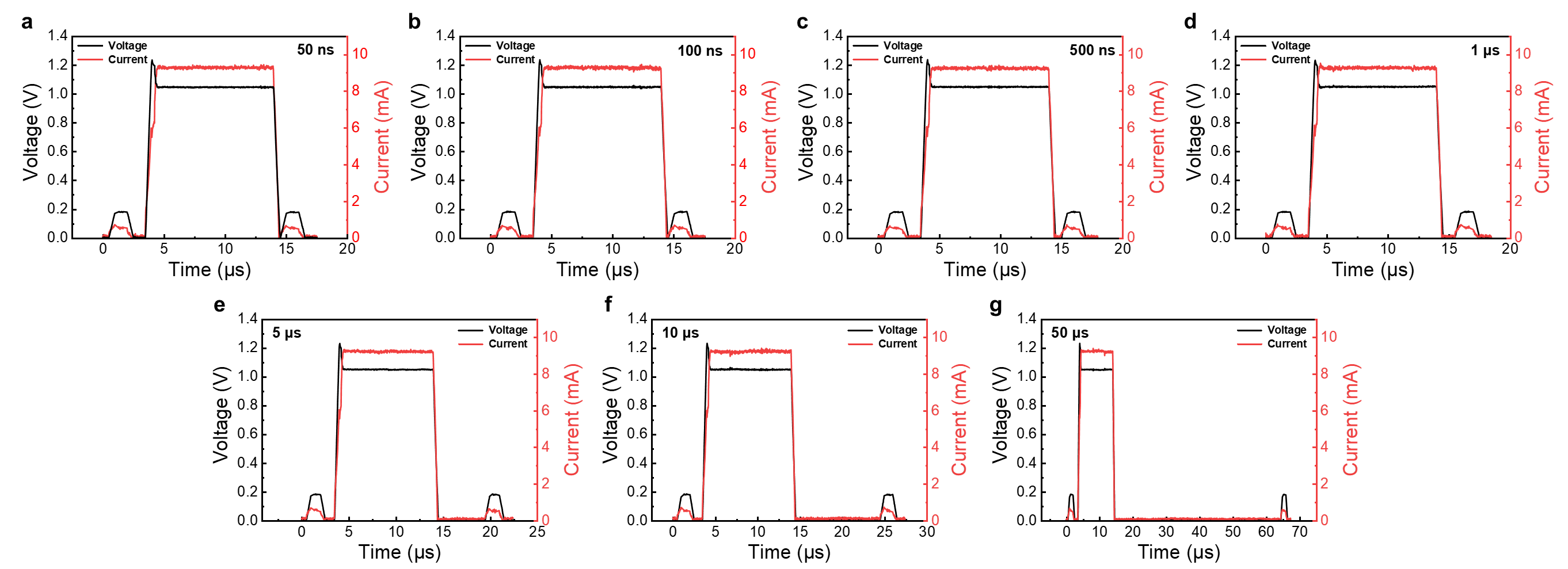


**Figure S4**. A result of recovery characteristics in various interval time ranges from 50 ns to 50 μs. Recovery interval of a) 50 ns, b) 100 ns, c) 500 ns, d) 1 μs, e) 5 μs, f) 10 μs, and g) 50 μs.

The experiment was carried out with the recovery interval reduced from 50 μs to 50 ns. Even at a 50 ns interval, the current value at the read voltage (0.1 V) before the on voltage pulse (2.0 V) and the current value at the read voltage after the on voltage pulse are equal, indicating that the operating characteristics do not degrade even when a short (≤ 50 ns) on voltage pulse is applied. Exactly 2.0 V of on voltage pulse was applied, but the actual measured voltage is around 1.2 V due to the voltage drop that occurs when the device is turned on at about 1.2 V.


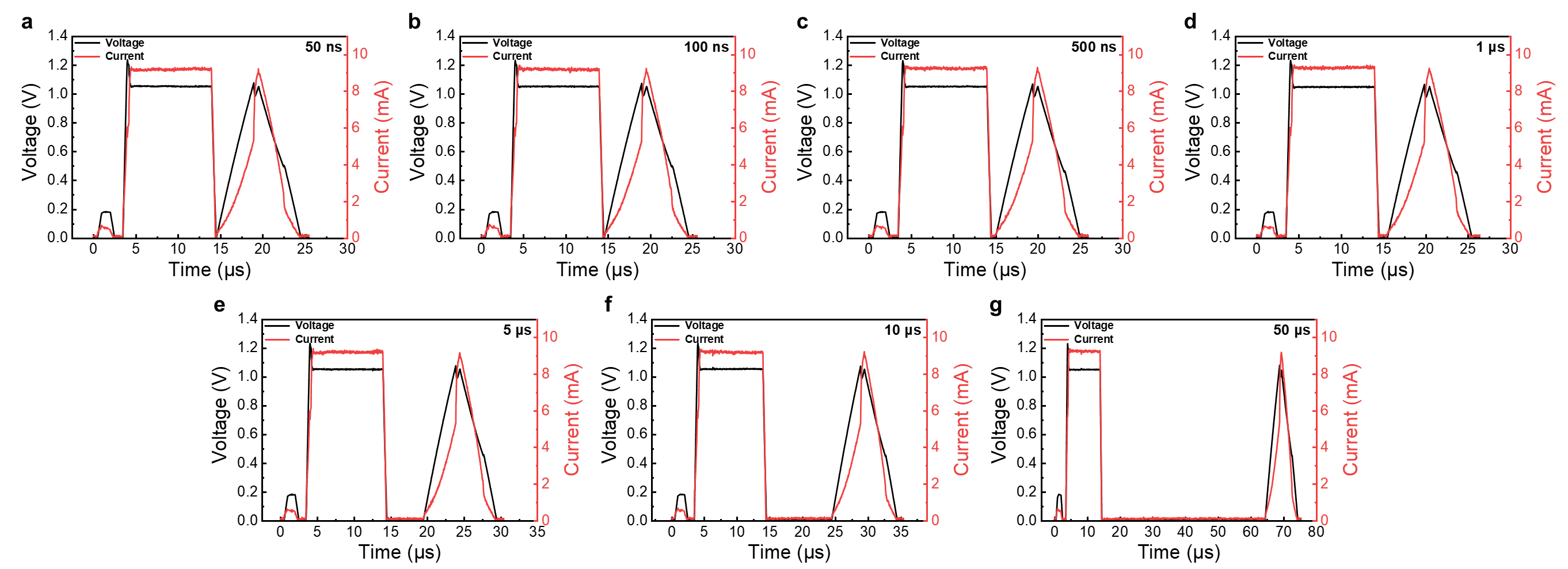


**Figure S5**. A result of wait time (drift-free) characteristics in various interval time range from 50 ns to 50 μs. Wait time interval of a) 50 ns, b) 100 ns, c) 500 ns, d) 1 μs, e) 5 μs, f) 10 μs, and g) 50 μs.

The experiment was carried out with the wait time interval reduced from 50 μs to 50 ns. Even at a 50 ns interval, the threshold voltage value at the on voltage pulse (2.0 V) and the threshold voltage value at the triangular voltage pulse after the on voltage pulse are equal, indicating that the device has a drift-free property even when a short (≤ 50 ns) on voltage pulse is applied. The recovery time and wait time experiments indicate that when manufactured as an array structure, such as 1S1R, using the ZnTe-based stack, it can operate stably even at high speeds (50 ns).


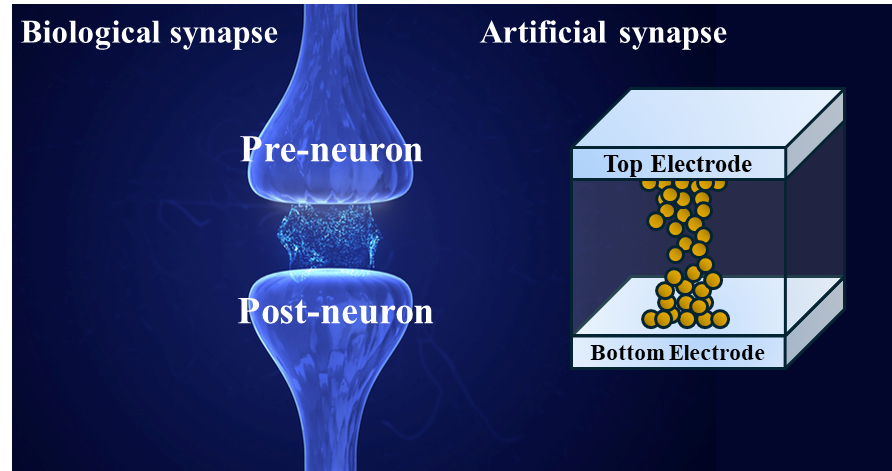


**Figure S6**. The contrast between artificial and biological synapses. Pre- and post-synaptic neurons are connected by synapses to form the biological nervous system. The memristor has the ability to imitate the biological synapse.


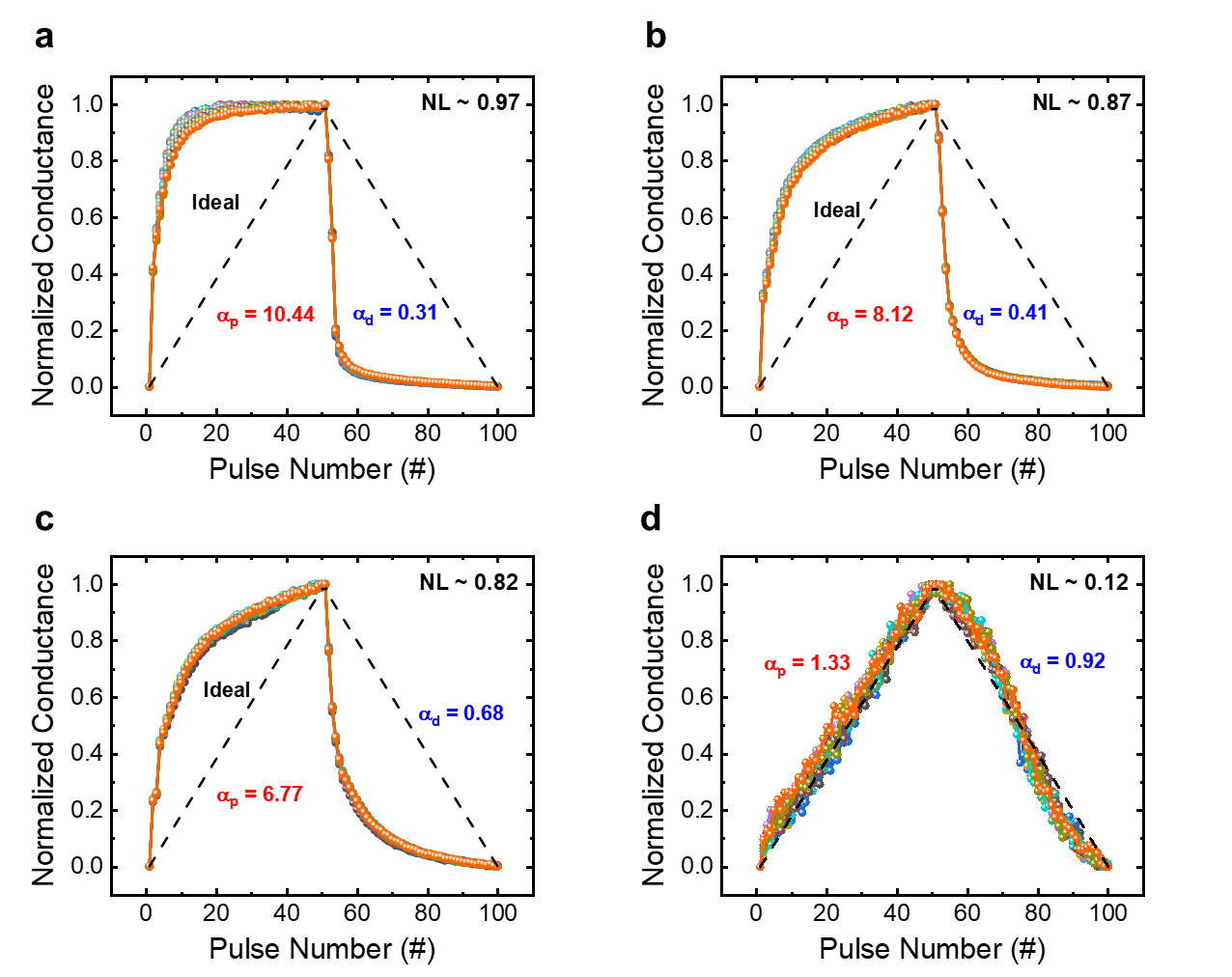


**Figure S7**. Results of potentiation and depression measured in the ZnTe-based device for 10 pulse cycles using the a), b), and c) identical pulse scheme, and d) incremental pulse scheme.


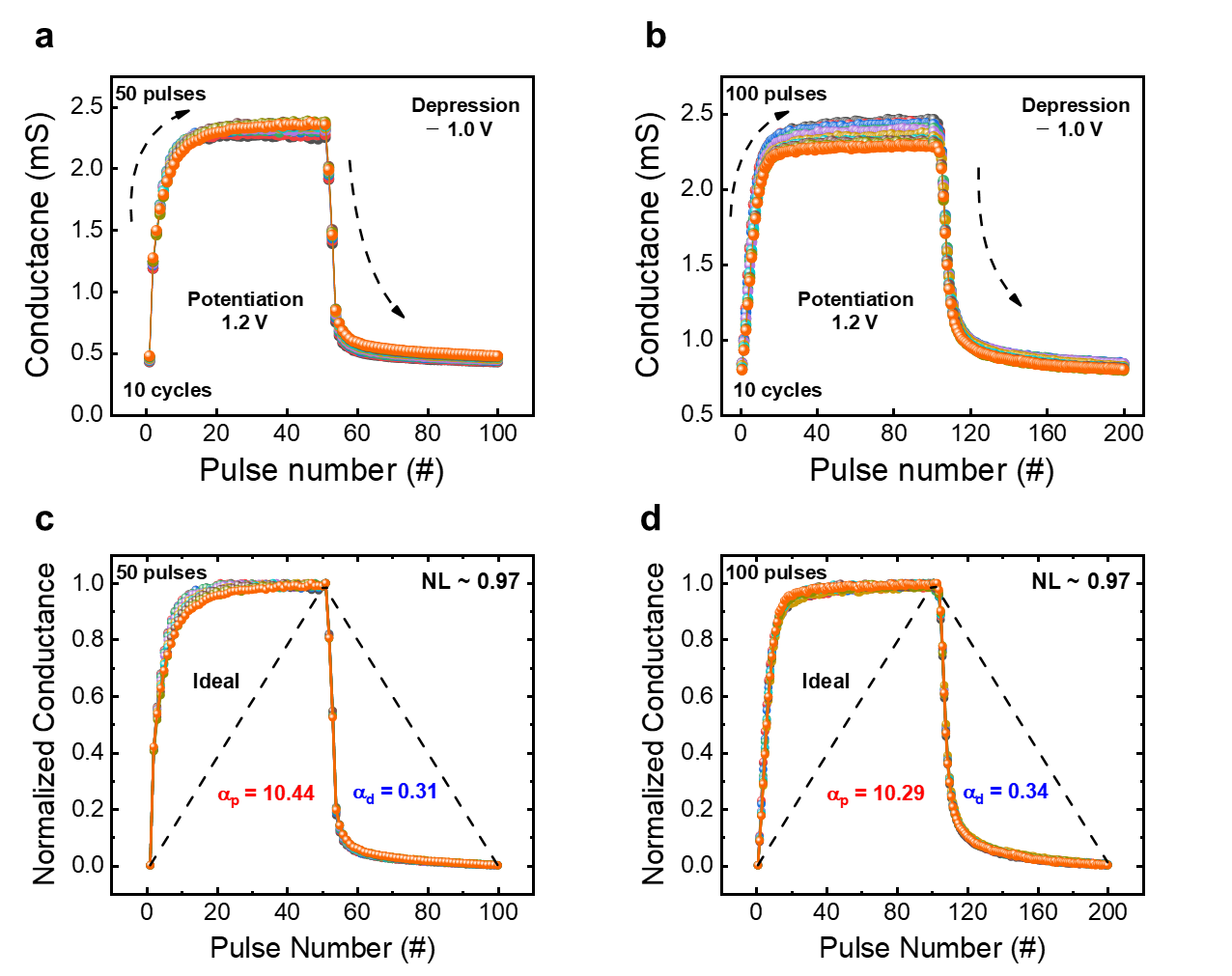


**Figure S8**. 10 cycles results of potentiation and depression measured in identical pulse for a) 50 pulses, and b) 100 pulses. Normalized conductance results of 10 cycles with nonlinearity factor in c) 50 pulses, and d) 100 pulses.

Multiple cycles of the potentiation and depression by varying the pulse amplitude from (0.8 to 1.2) and (−0.8 to −1.0) respectively. **Figures S7**a-c show potentiation voltages of 1.2 V, 1.0 V, and 0.8 V, respectively, and potentiation and depression voltages of −1.0 V, −0.9 V, and −0.8 V, respectively. In **Figure S7**d, the potentiation voltage gradually increased from 0.8 V to 1.2 V, depression voltage gradually dropped from −0.8 V to −1.0 V.

The nonlinearity (NL) and symmetric conductance changes were significantly enhanced, producing almost linear behavior, in contrast to the results attained by using identical pulses. To assess the degree of improvement quantitatively, we derived the NL factor. The maximum value of the conductance value difference normalized to the total plasticity during potentiation and depression is the NL value. The concept of NL is given as (1)^[1-2]^:

$NL=Max\left| G_{P}(n)-G_{D}(n) \right|, n=1, 2, 3, \cdot\cdot\cdot, 50$ (1)

where $G_{P}(n)$ and $G_{D}(n)$ are the normalized conductance value, which is from 0 to 1, and $n$ is the pulse number. By definition, it is most desirable for NL to be zero as shown in the linear dashed line (denoted as ideal) of **Figure S7** because linear weight update is the most ideal in hardware-based neuromorphic system development.

From (1), three separate identical and incremental pulses yielded NL values of 0.96, 0.84, 0.86, and 0.26, respectively. In other words, amplitude modulation gradually improves linearity and symmetry. Referring to the potentiation and depression conductance, it becomes evident that abrupt conductance change is present. To reduce this behavior, minimize abrupt changes in conductance, it is recommended to begin the process with a small voltage, reducing the NL.

In neuromorphic systems, the linearity of the potentiation and depression processes of the two-terminal memristor is crucial. These processes are related to the increase or decrease in the strength of the synaptic connection, and their linearity directly affects the learning ability of the neural network^[3]^. Based on this paper, we set the values of ${\alpha_{potentiation}(\alpha}_{p})$ and ${\alpha_{depression}(\alpha}_{d})$ as parameters applied when updating the synaptic weight, and the synaptic weight is updated using the following equation (2), (3):

$G$ = (($G_{LRS}^{\alpha}- G_{HRS}^{\alpha}$) × $\omega$ ＋ ${G_{HRS}^{\alpha} )}^{\alpha}$ if $\alpha\neq0$ (2)

$G$ = $G_{HRS}$× ${( G_{LRS} / G_{HRS})}^{\omega}$ if $\alpha＝ 0$ (3)

Here, $G_{LRS}$ refers to the conduction value of LRS, and $G_{HRS}$ refers to the conduction value of HRS. $\alpha$ is a parameter that reflects changes in potentiation and depression. When the values of ${\alpha_{potentiation}(\alpha}_{p})$ and ${\alpha_{depression}(\alpha}_{d})$ are close to 1, the learnable weight is more evenly distributed. Conversely, when the values of $\alpha_{p}$ and $\alpha_{d}$ differ significantly from 1, excessive weight updates may occur, leading to a reduced weight representation in this range. The values of $\alpha_{p}$ and $\alpha_{d}$ are closely correlated with the online neural network learning performance. The values of $\alpha_{p}$ and $\alpha_{d}$ in the identical pulse of our device are shown in **Figure S7** a-d respectively. This indicates that the MNIST pattern accuracy is less than 6% higher because the $\alpha_{p}$ and $\alpha_{d}$ values obtained through the incremental pulse are closer to 1.

On the other hand, if the number of pulses is increased from 50 to 100 steps, the change in conductance values decreases, as shown in **Figure S8** a, b, potentially increasing the learning rate. However, even with an increase in the number of pulses, if the P/D modification does not change completely linearly, the values of $\alpha_{p}$ and $\alpha_{d}$ do not change significantly, as shown in **Figure S8** c, d. There will be a limit to improving the learning rate due to the abruptly change between the initial conductance values. There was only a 0.37% difference between the actual MNIST pattern accrual and the results of 50 pulses and 100 pulses.

In conclusion, applying an intellectual pulse or incremental pulse scheme of different voltage magnitudes, or setting an appropriate number of pulses, is necessary to improve the learning rate in P/D. However, as the pulse configuration varies, trade-off such as decreased learning speed and increased power consumption should be considered. Plus, like our device, the performance improvement may be insufficient.


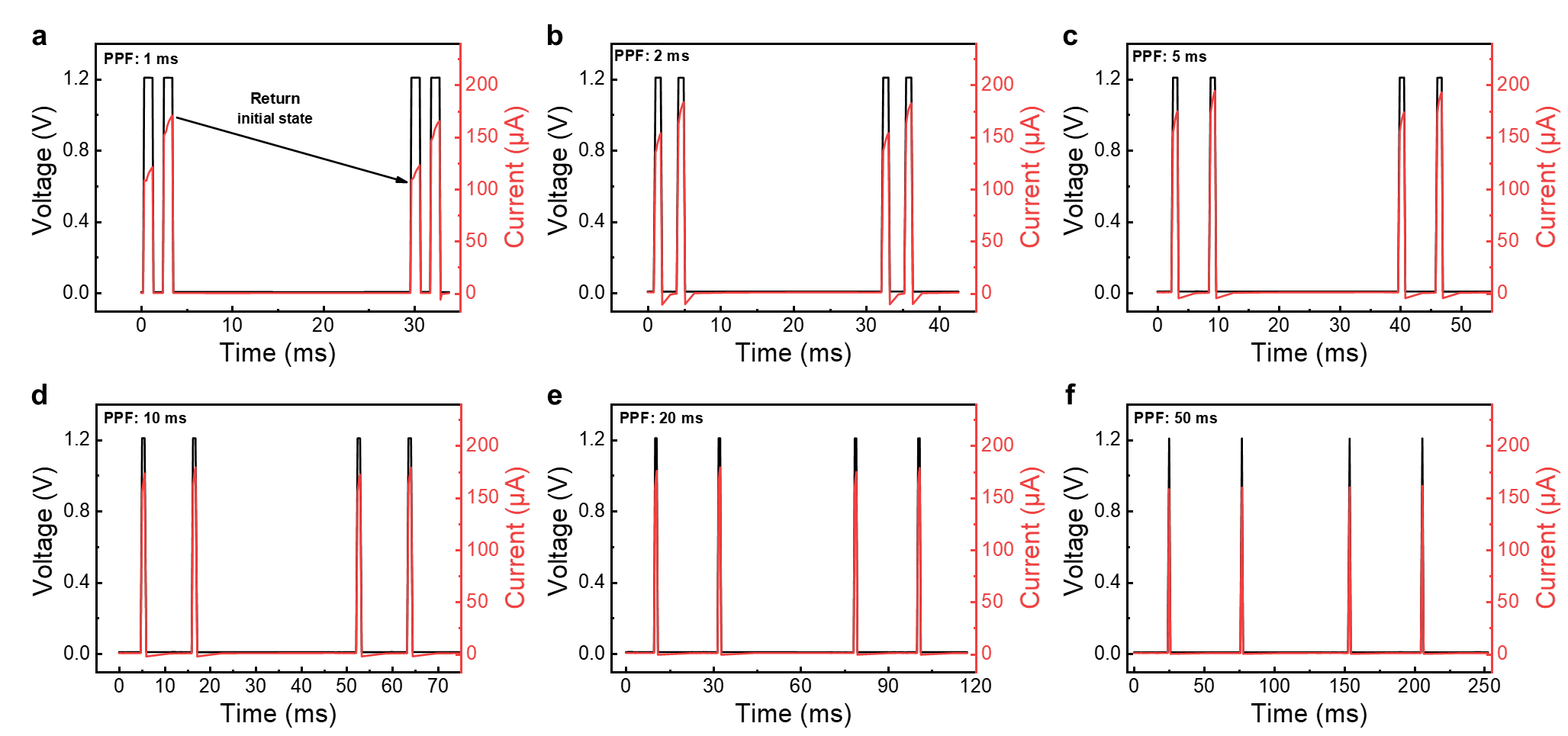


**Figure S9**. A result of PPF measurements in various interval time range from 1 ms to 50 mA. PPF interval of a) 1 ms, b) 2 ms, c) 5 ms, d) 10 ms, e) 20 ms, f) 50 ms.


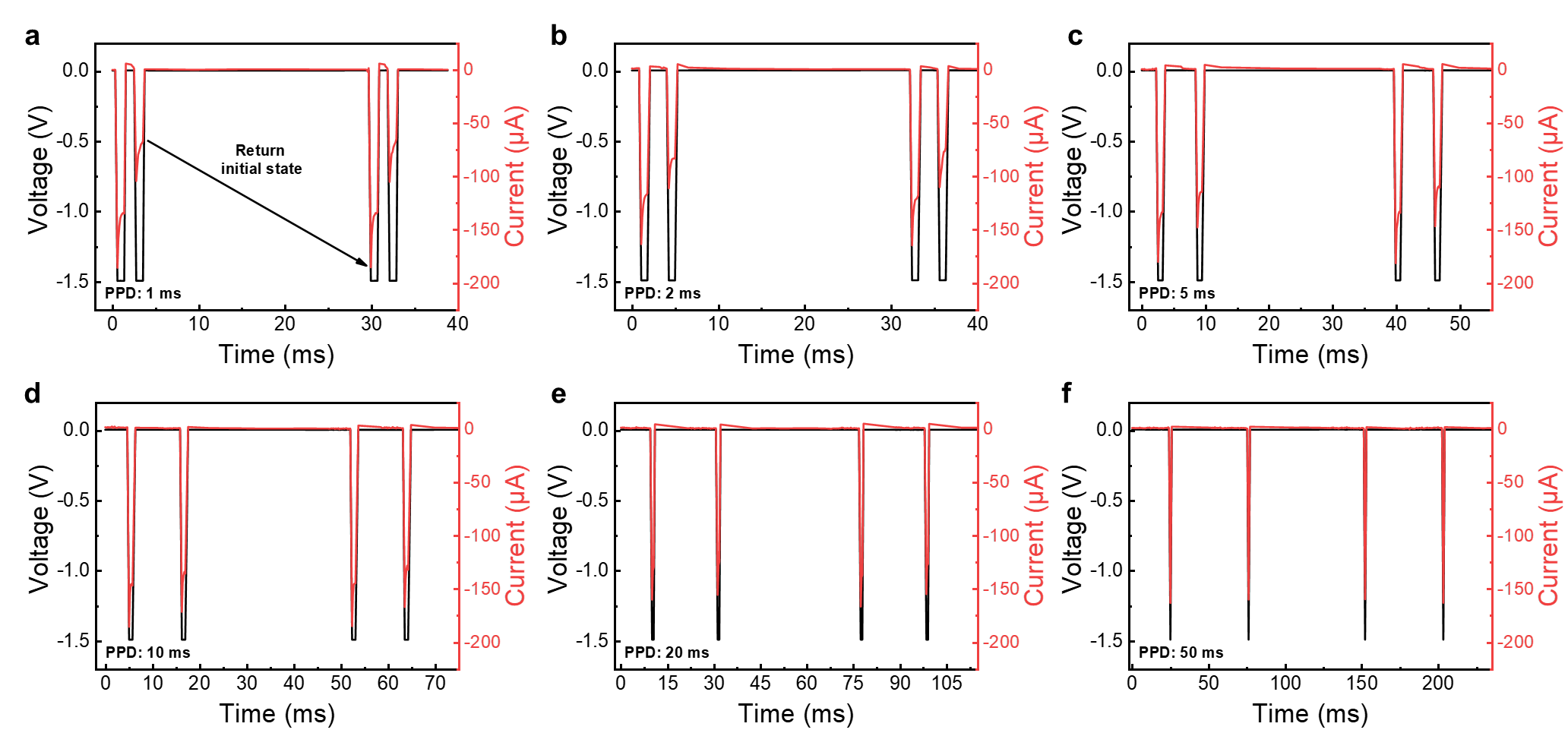


**Figure S10**. A result of PPD measurements in various interval time range from 1 ms to 50 mA. PPD interval of a) 1 ms, b) 2 ms, c) 5 ms, d) 10 ms, e) 20 ms, f) 50 ms.


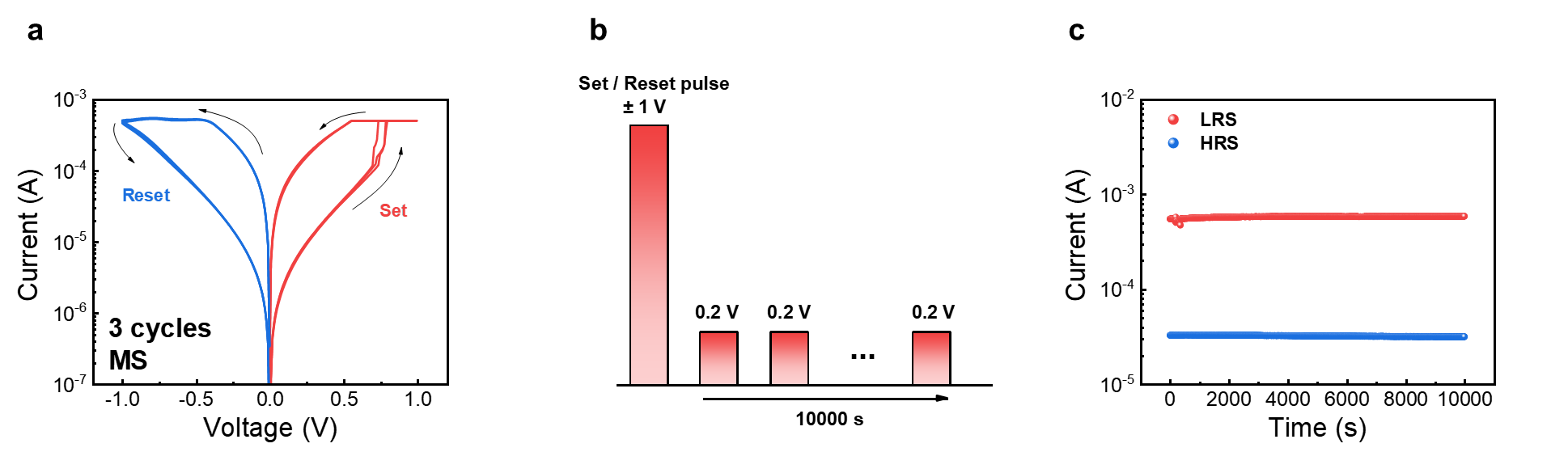


**Figure S11**. a) I-V curves before measure the retention. b) Pulse scheme of the retention. c) A result of retention measurements in memory switching region for 10,000 s.


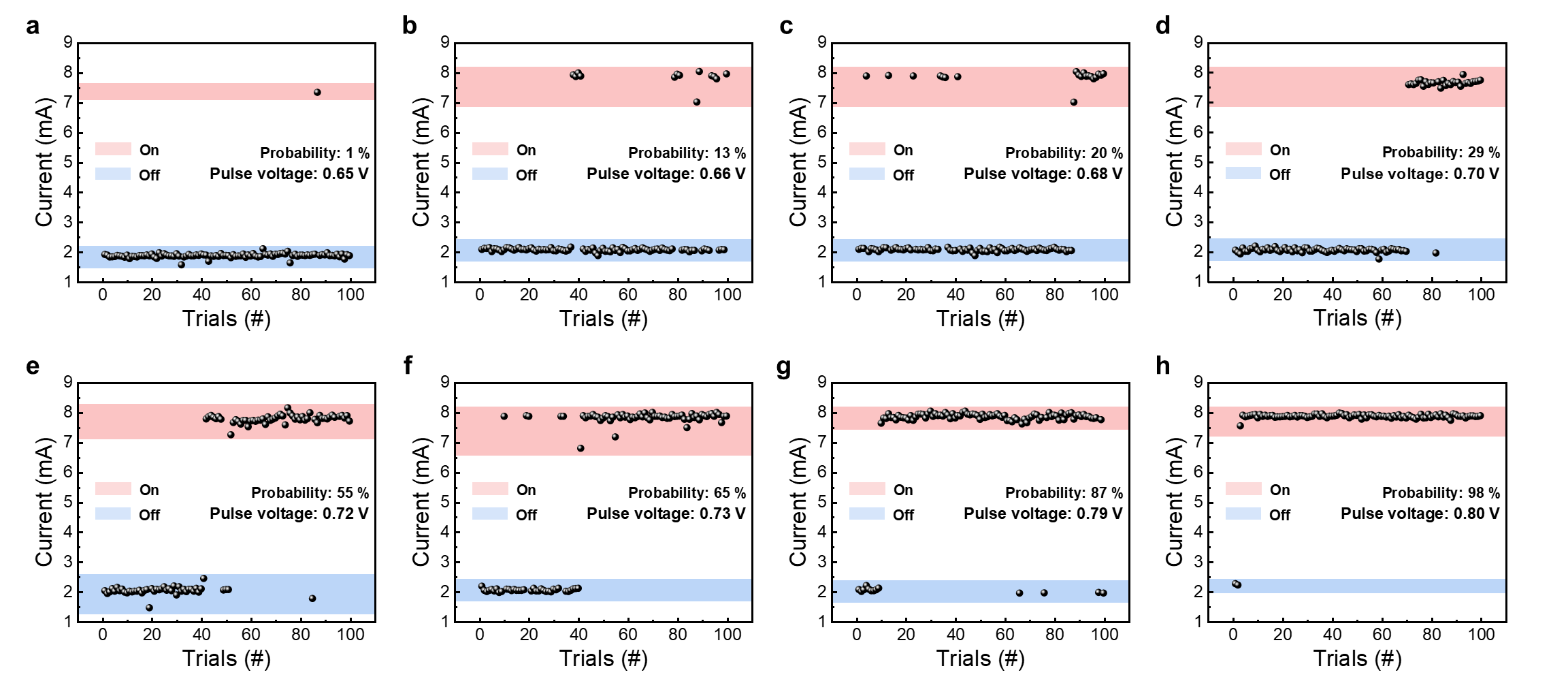


**Figure S12**. Stochastic response (result of switching probability) of the device based on fixed pulse width (50 μs) and various voltage amplitude in a) 0.65 V, b) 0.66 V, c) 0.68 V, d) 0.70 V, e) 0.72 V, f) 0.73 V, g) 0.79 V, and h) 0.80 V.


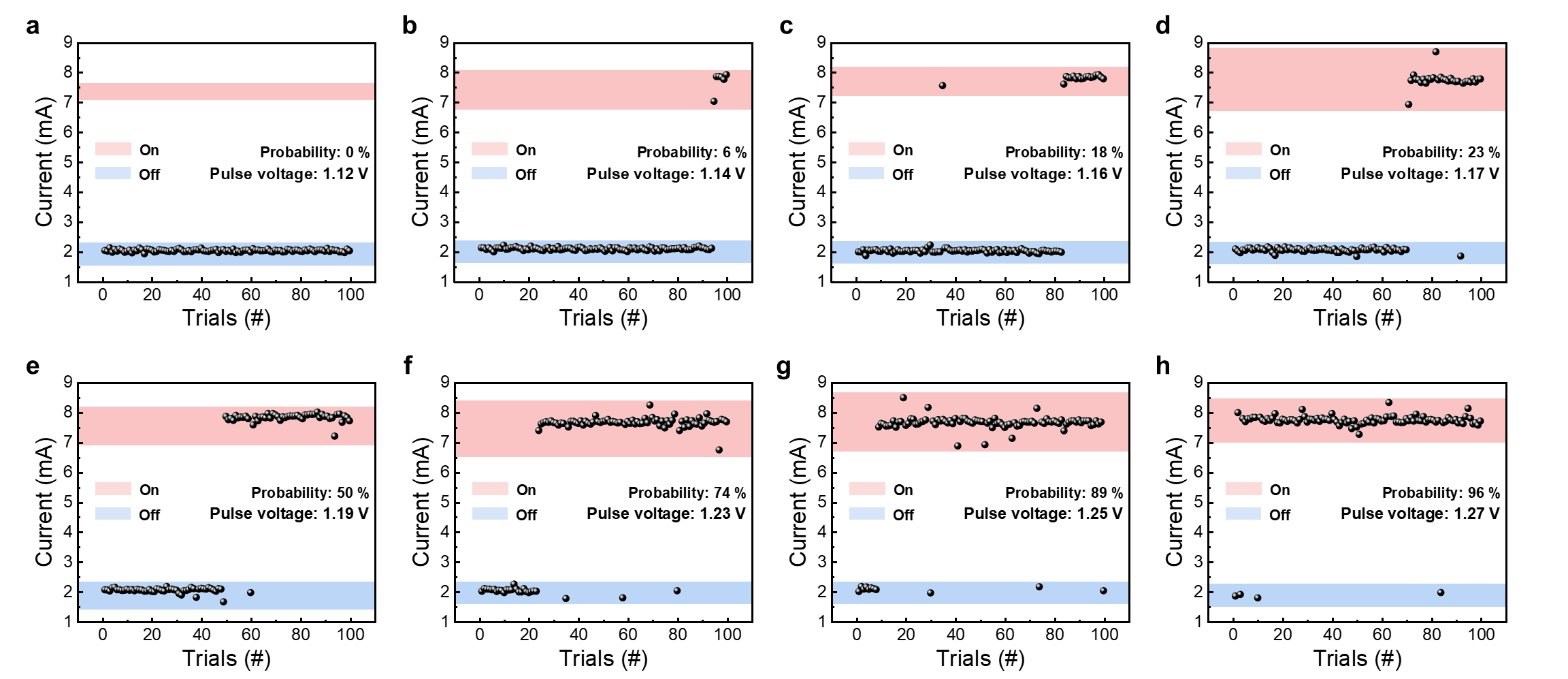


**Figure S13**. Stochastic response (result of switching probability) of the device based on fixed pulse width (5 μs) and various voltage amplitude in a) 1.12 V, b) 1.14 V, c) 1.16 V, d) 1.17 V, e) 1.19 V, f) 1.23 V, g) 1.25 V, and h) 1.27 V.


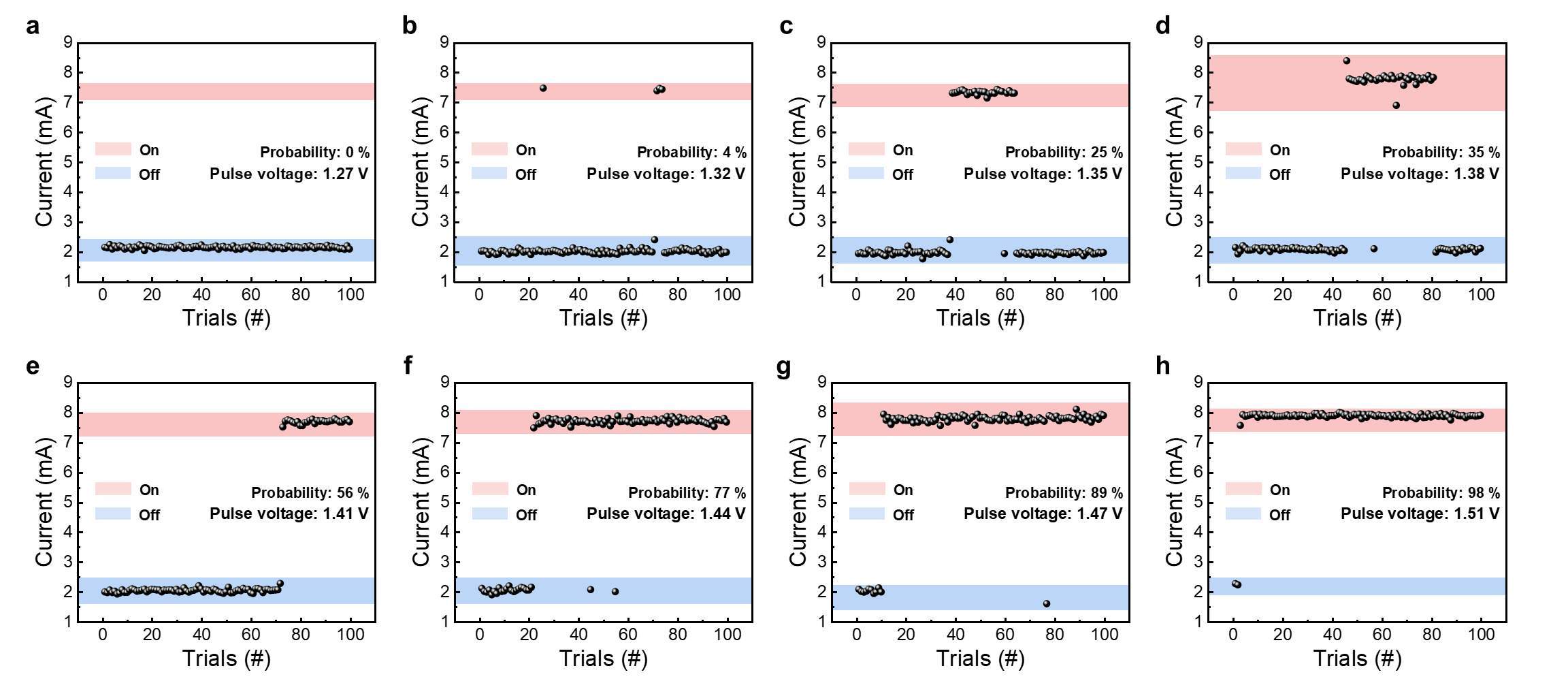


**Figure S14**. Stochastic response (result of switching probability) of the device based on fixed pulse width (0.5 μs) and various voltage amplitude in a) 1.27 V, b) 1.32 V, c) 1.35 V, d) 1.38 V, e) 1.41 V, f) 1.44 V, g) 1.47 V, and h) 1.51 V.

Due to the threshold switching behavior, which only maintains the "on" state during the pulse applied, a reset pulse is unnecessary. Applying a fixed pulse amplitude multiple times can induce an "on" state during a specific trial. The on-state is defined as the current value is greater than 7 mA. After 100 pulses were applied, the switching probability was calculated as (4):

Switching probability = $\frac{N_{on}}{N_{num}}\times100 (\%)$ (4)

where, $N_{on}$ is Total number of on-state, $N_{num}$ is total number of pulse (trials). The pulse amplitude was gradually increased at each pulse width to calculate the switching probability using (4). The transition from stochastic response to deterministic response is more likely to occur the larger the pulse amplitude across all pulse widths.


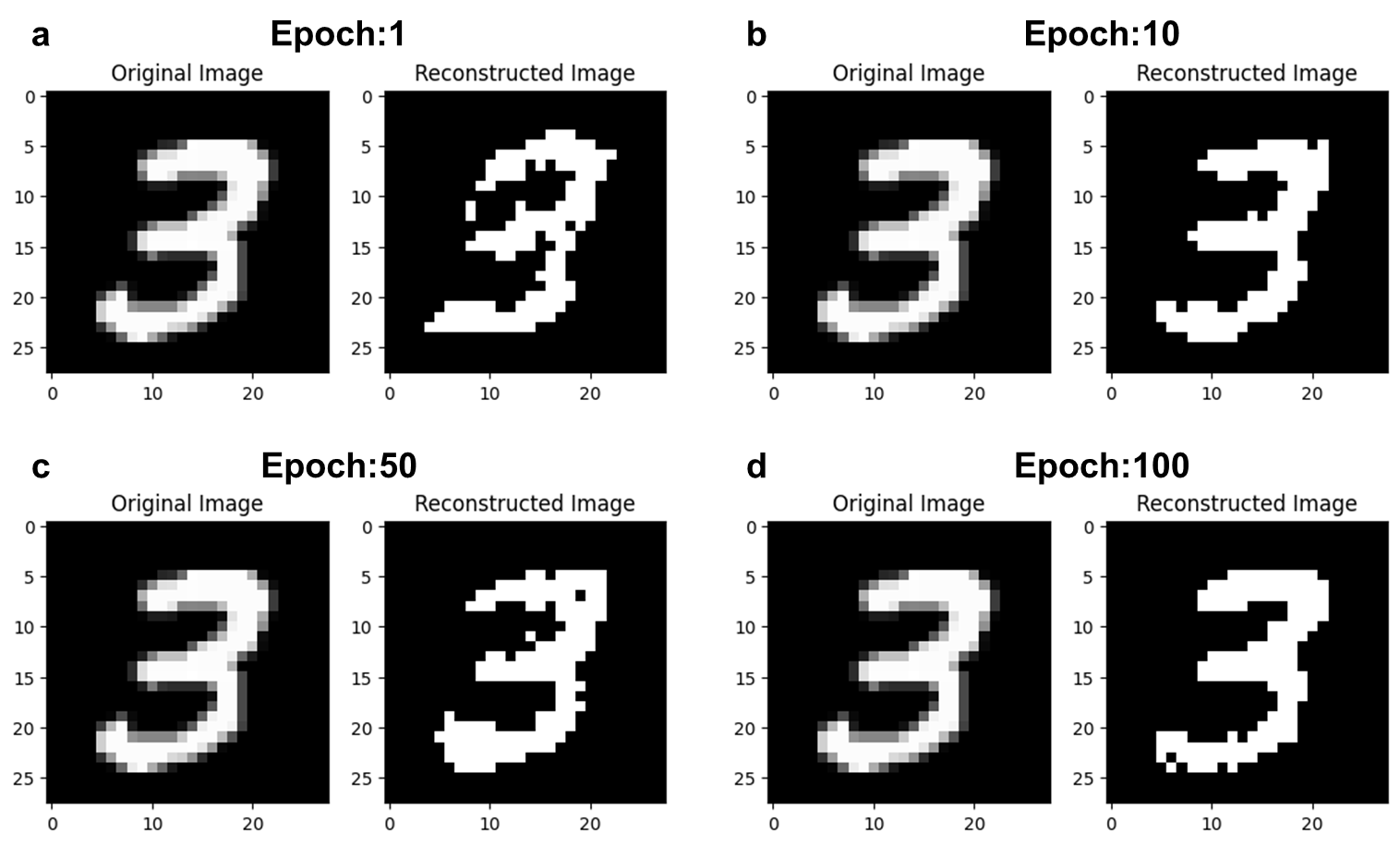


**Figure S15**. The alternation of reconstructed MNSIT image in various epoch in a) 1, b) 10, c) 50, and d) 100.

The original data can be restored in RBM model with key feature of original data. The degree to which the original data is restored will be an essential factor in evaluating the effectiveness of the RBM model. Thus, using the ZnTe-based memristor, a Python code was created to visually evaluate the performance of the RBM model. As the epoch goes on, **Figure S15**a-d shows how the "Reconstructed Image" on the right side of each figure resembles the "Original Image," which is the original data. The result demonstrates that this ZnTe-based device can function as component of an RBM model.


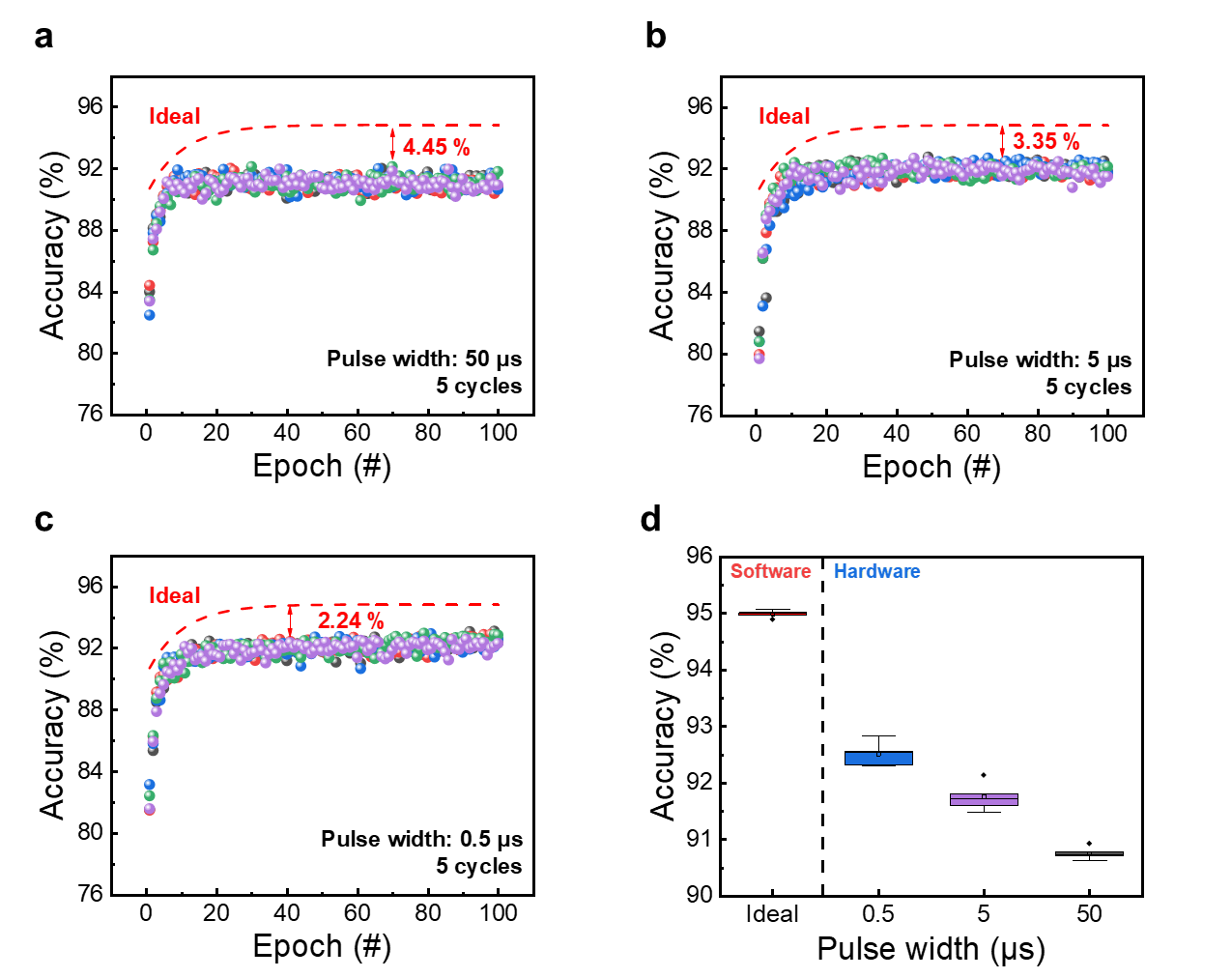


**Figure S16**. The comparison of RBM model performance based on three sigmoid function and general logistic sigmoid function attained by various pulse width. The result of MNIST classification accuracy 5 cycles about three fixed pulse width a) 50 μs, b) 5 μs, and c) 0.5 μs. d) comparison of accuracy in software (ideal, non-modulation sigmoid function) and hardware (three distinct sigmoid function).

Reference

[1] I.-T. Wang, C.-C. Chang, L.-W. Chiu, T. Chou, T.-H. Hou, *Nanotechnology* **2016**, *27*, 365204.

[2] S. Choi, J. Yang, G. Wang, *Advanced Materials* **2020**, *32*, 2004659.

[3] J. Park, M. Kwak, K. Moon, J. Woo, D. Lee, H. Hwang, *IEEE Electron Device Letters* **2016**, *37*, 1559.
